# Role of TRPM8 in switching between fever and hypothermia in adult mice during endotoxin-induced inflammation

**DOI:** 10.1016/j.bbih.2021.100291

**Published:** 2021-06-30

**Authors:** Chinatsu Shiraki, Ririka Horikawa, Yuzuki Oe, Momoka Fujimoto, Kaho Okamoto, Erkin Kurganov, Seiji Miyata

**Affiliations:** Department of Applied Biology, Kyoto Institute of Technology, Matsugasaki, Sakyo-ku, Kyoto, 606-8585, Japan

**Keywords:** Fever, Hypothermia, Sepsis, TLR4, TLR2, LPS, Zymosan, IL-1β, Inflammation, Brain

## Abstract

Transient receptor potential melastatin 8 (TRPM8) functions in the sensing of noxious and innocuous colds; however, its significance in pathogen-induced thermoregulation remains unclear. In the present study, we investigated the role of TRPM8 in the regulation of endotoxin-induced body temperature control. The peripheral administration of low-dose lipopolysaccharide (LPS) at 50 ​μg/kg generated fever in wild-type (WT) mice, whereas it caused hypothermia in TRPM8 knockout (KO) animals. LPS-induced sickness responses such as decrease in body weight, and food and water intake were not different between WT and TRPM8 KO mice. TRPM8 KO mice exhibited more severe hypothermia and lower locomotor activity following the peripheral administration of high-dose LPS at 5 ​mg/kg compared with WT ones. An intracerebroventricular (i.c.v.) injection of either LPS at 3.6 ​μg/kg or interleukin-1β at 400 ​ng/kg elicited hypothermia in TRPM8 KO mice, in contrast to fever in WT animals. The peripheral administration of zymosan at 3 ​mg/kg also induced hypothermia in contrast to fever in WT mice. An i.c.v. injection of prostaglandin E_2_ at 16 or 160 ​nmol/kg induced normal fever in both WT and TRPM8 KO mice. Infrared thermography showed significant decline of the interscapular skin temperature that estimates temperature of the brown adipose tissue, regardless of no alteration of its temperature in WT animals. Fos immunohistochemistry showed stronger Fos activation of hypothalamic thermoregulation-associated nuclei in TRPM8 KO mice compared with WT animals following the peripheral administration of low-dose LPS. Therefore, the present study indicates that TRPM8 is necessary for switching between fever and hypothermia during endotoxin-induced inflammation.

## Highlights

• Low-dose LPS (ip) caused hypothermia in TRPM8 KO animals versus fever in WT ones.
• TRPM8 KO mice showed severe hypothermia by high-dose LPS (ip).
• Central LPS injection elicited hypothermia in TRPM8 KO mice versus fever in WT ones.
• Low-dose LPS (ip) caused decrease in BAT temperature.
• Low-dose LPS (ip) caused more hypothalamic Fos expression in TRPM8 KO mice than WT.

## Graphical abstract

**Figure:**
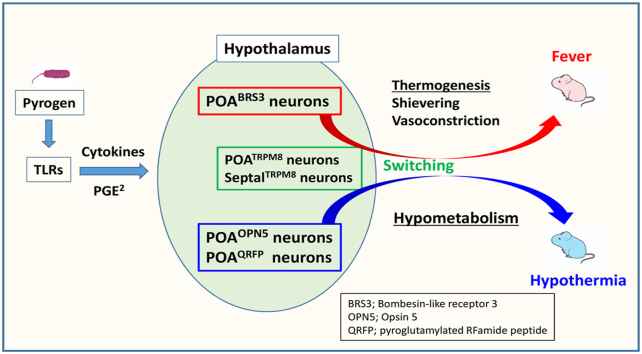

## 1 Introduction

The recognition of pathogen infections is one of the most important mechanisms of innate immunity for eliminating pathogens and the first line of host defense against infection (Thaiss et al., 2016). Several families of pattern recognition receptors are responsible for detecting the pathogen-associated molecular patterns (PAMPs) of many pathogens (Beutler, 2009). Toll-like receptors (TLRs) are the most important and widely studied pattern recognition receptors (Lemaitre et al., 1996; Beutler, 2009). The infection-induced activation of immune cells via TLRs leads to a broad spectrum of sickness symptoms, such as body temperature changes, nausea, decreased appetite, malaise, and fatigue, which are adaptive responses to promote animal survival (McCusker and Kelley, 2013).

Fever is generated by integrated physiological and neuronal responses that confer a survival benefit against infection (Vaughn et al., 1974; Kluger, 1979). The inhibition of fever with antipyretic drugs was previously reported to increase mortality in humans infected with influenza virus (Schulman et al., 2005). Fever enhances immunoprotective mechanisms in both the innate and adaptive immune systems, such as the acceleration of cytokine production and the cellular cytotoxic activity of dendritic cells, natural killer cells, macrophages as well as signaling by and the differentiation of T cells (Evans et al., 2015). Fever also results in an increased metabolic rate, with only a 1 ​°C increase in body temperature elevating the metabolic rate by approximately 10% (Kluger, 1979).

Hypothermia occurs in the most severe cases of systemic inflammatory syndromes instead of fever and is regarded as an adaptive host strategy to attenuate the harmful effects caused by the overshoot of inflammatory responses (Romanovsky et al., 1996). Hypothermia suppresses endotoxin increases, abdominal organ dysfunction, hypotension, and mortality in animals injected with *Escherichia coli* (Liu et al., 2012). The intraperitoneal administration of the TLR4 agonist, lipopolysaccharide (LPS), was shown to induce fever or hypothermia in mouse depending on dose: a low dose of 50–100 ​μg/kg induced fever, whereas a high-dose of more than 2.5 ​mg/kg caused hypothermia (Oka et al., 2003; Garami et al., 2018). The intraperitoneal administration of a low dose of the TLR2 agonist zymosan of 1–10 ​mg/kg induced fever (Bastos-Pereira et al., 2014), whereas a high-dose of more than 50 ​mg/kg caused hypothermia (Takagi et al., 2020). Therefore, the two thermoregulatory responses are complementary strategies of survival in infection-induced inflammation.

Prostaglandin E_2_ (PGE_2_), a lipid mediator, plays a key role in eliciting fever or hypothermia by acting on prostaglandin EP receptors in the preoptic area (POA) of the hypothalamus (Garami et al., 2018). Recent studies reported that the medial and lateral POA is an important thermoregulatory center in the brain (Hrvatin et al., 2020; Takahashi et al., 2020; Zhang et al., 2020). The specialized population of these POA neurons elicits a long-lasting hypothermic and low metabolic state by acting on the dorsomedial hypothalamic nucleus (DMH) (Hrvatin et al., 2020; Takahashi et al., 2020; Zhang et al., 2020). The circumventricular organs (CVOs), which are characterized by fenestrate capillaries or the lack of the blood-brain barrier (McKinley et al., 2003; Roth et al., 2004; Siso et al., 2010; Miyata, 2015). Moreover, TLR2 and TLR4 are expressed at microglia/macrophages and astrocytes/tanycytes, respectively, in the CVOs (Chakravarty and Herkenham, 2005; Nakano et al., 2015; Muneoka et al., 2019; Murayama et al., 2019). The selective deletion of cyclooxygenase-2 in brain endothelial cells has been shown to attenuate the fever response of mice to peripheral administration of LPS and interleukin-1β (IL-1β) (Wilhelms et al., 2014). Whole deletion of the PGE_2_ prostaglandin E receptor 3 eliminates the fever response, whereas its deletion of peripheral nervous system has no effect (Eskilsson et al., 2021). Moreover, deletion of the IL-1 receptor 1 or interleukin-6 (IL-6) receptor α on brain endothelial cells attenuates the fever response, although its deletion on peripheral nerves has no change (Eskilsson et al., 2021). Thus, cytokines or PGE_2_ action to brain endothelial cells is critical for endotoxin-induced fever.

Transient receptor potential melastatin 8 (TRPM8) is activated by noxious and innocuous cooling (McKemy et al., 2002; Peier et al., 2002), and responds to alterations in temperature decreases regardless of whether the temperature is above the activation threshold (Ran et al., 2016; Yarmolinsky et al., 2016). TRPM8 is also activated by chemical regents such as menthol and icilin (Zu et al., 2020). Moreover, TRPM8 knockout (KO) mice have decreased cold responses in neurons of the dorsal root ganglion and severe behavioral deficits in response to cold stimuli (Bautista et al., 2007; Dhaka et al., 2007). Moreover, TRPM8 KO mice generates obesity and metabolic dysfunction when they were kept under cold ambient temperature (Reimúndez et al., 2018). Apart from cold sensation, recently, it is reported that TRPM8 is also required for the detection and perception of skin warming (Paricio-Montesinos et al., 2020). Although TRPM8 has been shown to be obviously participate in peripheral thermosensation, the significance of TRPM8 in endotoxin-induced body temperature regulation remains completely unknown.

To directly address this issue, the present study aimed to elucidate whether TRPM8 is involved in switching of endotoxin-induced fever and hypothermia. Currently, limited information is available on the switching mechanism between fever and hypothermia. Our data found that hypothermia was induced in TRPM8 KO mice, in contrast to fever in wild-type (WT) animals, by the peripheral administration of low-dose LPS or zymosan. A similar hypothermic response was observed in TRPM8 KO mice following the intracerebroventricular (i.c.v.) injection of either LPS or IL-1β despite normal fever generation with the i.c.v. injection of PGE_2_. Moreover, the peripheral administration of high-dose LPS induced prolonged severe hypothermia in TRPM8 KO mice. The infrared thermography revealed that the peripheral administration of low-dose LPS caused remarkable decrease in temperature of the interscapular skin surface that is compatible to that of the brown adipose tissue (BAT). The number of Fos-expressing neurons in hypothalamic thermoregulation-associated regions was significantly higher in TRPM8 KO mice than in WT animals after the peripheral administration of low-dose LPS. These results indicate that central TRPM8 plays a crucial function in switching between fever and hypothermia during endotoxin-induced inflammation, and propose a new aspect for infection-induced body temperature regulation in human.

## 2 Materials and methods

### 2.1 Animals

Adult male C57BL/6 ​J mice (8–10 weeks old) were obtained from Japan SLC Inc. (Hamamatsu, Japan). TRPM8 KO mice were originally generated by Dr. A. Patapoutian at the Howard Hughes Medical Institute, the Scripps Research Institute on the C57BL/6 background (Dhaka et al., 2007) and subsequently bred and supplied by Dr. M. Tominaga at the National Institute of Physiological Sciences. TRPM8 KO mice were genetically engineered by knocking in EGFP followed by a SV40polyA at frame of start codon of TRPM8 and the SV40polyA tail prevented transcription of TRPM8. TRPM8 KO mice were genotyped by either PCR or immunohistochemistry to confirm the lack of the TRPM8 allele and maintained in our institute under specific pathogen-free conditions. Animals were housed in a colony room with an ambient temperature of 25 ​± ​0.5 ​°C and a 12-hr light/dark cycle; lights on at 7:00 and lights off at 19:00, and were given *ad libitum* access to commercial chow and tap water. In some experiment, changes in body weight, and food and water intake were 24 ​h before and after the administration of low-dose LPS at 50 ​μg/kg. All experiments were performed in accordance with the Guidelines laid down by the NIH and Proper Conduct of Animal Experiments Science Council of Japan. The experimental protocol was approved by the Animal Ethics Experimental Committee of the Kyoto Institute of Technology (No. 100170, 100173).

### 2.2 Administration of LPS, zymosan, IL-1β, and PGE_2_

A stock solution of LPS (1 ​mg/ml; Sigma-Aldrich, 055: type B5), zymosan A from *Saccharomyces cerevisiae* (FUJIFILM Wako Chemical Pure Corporation, Osaka, Japan), IL-1β (R&D systems, Minneapolis, MN), and PGE_2_ (Cayman Chemical, Ann Arbor, MI) were dissolved in pyrogen-free physiological saline (Otsuka Pharmaceutical Co., Ltd.) and stored at −80 ​°C. They were diluted with pyrogen-free physiological saline prior to use.

The peripheral administration of endotoxins, mice were intraperitoneally administered an aliquot of pyrogen-free physiological saline containing low-dose LPS (50 ​μg/kg, 1.25 μg/300 ​μl), high-dose LPS (5 ​mg/kg, 125 μg/300 ​μl), or zymosan (3 ​mg/kg, 75 μg/200 ​μl). In the i.c.v. administration, a stainless steel cannula (25-gauge) was implanted in each mouse under anesthesia with isoflurane so that the tip was positioned in the lateral cerebral ventricle using a standard stereotaxic technique (Paxinos and Franklin, 2007); 0.3 ​mm anteroposterior and 1.0 ​mm lateral to the bregma and 2.5 ​mm dorsoventral below the skull. A G2 E-mitter transponder (Starr Life Sciences, Oakmont, PA) was implanted one week after cannula surgery. Freely moving mice received an i.c.v. injection (3 ​μl, 0.5 ​μl/min) of LPS (3.6 ​μg/kg, 90 ng/3 ​μl), IL-1β (400 ​ng/kg; 10 ng/3 ​μl), low-dose PGE_2_ (16 ​nmol/kg, 0.4 nmol/3 ​μl), high-dose PGE_2_ (160 ​nmol/kg, 4 nmol/3 ​μl), or pyrogen-free physiological saline using a Model EP-1000 ​E administration pump (Melquest, Toyama, Japan).

### 2.3 Measurement of body temperature and locomotor activity

Under anesthesia with isoflurane, the G2 E-mitter transponder was implanted intraperitoneally to record changes in core body temperature and gross locomotor activity. After 1-week recovery period, mouse body temperature was measured at an ambient temperature of 25 ​°C ​± ​0.5 ​°C under a 12-hr light/dark cycle (lights on at 7:00 and lights off at 19:00). LPS-induced hypothermia in mice is observed at subneutral or cool ambient temperature, but is not seen at neutral temperature of about 29/30 ​°C (Rudaya et al., 2005; Gordon, 2012; Liu et al., 2012; Garami et al., 2018), and therefore, ambient temperature was set at 25 ​°C. Intraperitoneal and i.c.v. administration protocols were initiated at 11:00. Abdominal temperature was measured by biotelemetry at 5-min intervals, except at 1-min intervals for the PGE_2_ treatment, over a period of 12 ​h before and 24 ​h after the treatment. Data were acquired and fed to a computer using VitalView software (VitalView series 4000). The baseline temperature was calculated as the mean core body temperature in each group at 10:45–11:00 according to the previous reports (Oka et al., 2003; Song et al., 2016; Reimúndez et al., 2018). The temperature index (°C ​× ​hr or °C ​× ​min) was calculated as the area under the temperature curve according to the baseline temperature before the treatment.

### 2.4 Measurement of skin surface temperature

The recording of surface temperature was made by infrared imaging, as previously reported (Marks et al., 2009; Vianna and Carrive, 2012). The mice were shaved between the scapulae and over the lumbar back regions to expose the skin 3 days before the measurement. Mice were kept in the special round cage (150 ​mm diameter x 200 ​mm height) and were recorded with a highly sensitive infrared camera (FLIR C5; FLIR Systems AB, Täby, Sweden) positioned 35 ​cm above the floor of the round cage. The temperature of the scapulae (TiScap) and lumbar back (TBack) was measured with 30 ​min interval after the intraperitoneal administration of 50 ​μg/kg LPS. The camera has a thermal sensitivity of ~0.07 ​°C at 25 ​°C and spatial resolution of 160 ​× ​120 (19,200) pixels. The surface temperature was calculated using linear temperature measurer in FLIR Tool ​+ ​software (FLIR Systems AB). The software detects within the ellipse 2 ​cm in major axis and 1 ​cm in minor axis and calculates mean surface temperature. The baseline temperature was obtained mean surface temperature in each group 30 ​min before the administration of LPS. The change in surface temperature was calculated by the surface temperature at a given time point minus the baseline surface one.

### 2.5 Immunohistochemistry

Mice were perfused intracardially with PBS (pH 7.4) containing 0.1% trisodium citrate dihydrate followed by cold 4% paraformaldehyde (PFA) in 0.1 ​M phosphate buffer (PB; pH 7.4) under deep anesthesia with isoflurane. Fixed brains were cryoprotected by 30% sucrose in phosphate-buffered saline (PBS; pH 7.4) and frozen quickly in Tissue-Tek OCT compound (Sakura Finetechnical, Tokyo, Japan). Sections were obtained by a coronal cut on a cryostat (Leica, Wetzlar, Germany) at a thickness of 30 ​μm. In immunofluorescent staining, a standard technique was performed on free-floating sections as described in our previous study (Furube et al., 2018). In brief, sections were washed with PBS and treated with 25 ​mM glycine in PBS for 20 ​min to quench the remaining fixative aldehyde. Sections were preincubated with 5% normal goat serum (NGS) in PBS containing 0.3% Triton X-100 (PBST) at 4 ​°C for 24 ​h and then incubated with the primary antibody in PBST containing 1% NGS at 4 ​°C for 72 ​h. The following primary antibodies were used: a rabbit polyclonal antibody against Fos (Cat. No. sc-52, Santa Cruz Biotechnology, Santa Cruz, CA; dilution 1:3000) or TLR4 (SPC-200, StressMarq, Victoria, Canada, dilution 1:200). After several washes with PBST, they were further incubated with an Alexa 488-conjugated secondary goat antibody (Jackson ImmunoResearch, dilution 1:400). Regarding nuclear staining, sections were incubated with 4′,6-diamidino-2-phenylindole dihydrochloride solution (Dojindo, Kumamoto, Japan; dilution 1:1000).

### 2.6 Fluorescent microscopic observations

In fluorescent microscopic observations, coverslips were sealed with Vectashield (Vector Labs, Burlingame, CA) and observations were performed using a fluorescent microscope (AxioScope. 5, Carl Zeiss, Oberkochen, Germany). We selected at least 5 sections per animal from the POA (between the bregma 0.26 and 0.74 ​mm), LS (between the bregma 1.02 and 0.38 ​mm), paraventricular nucleus (PVN; between the bregma −0.74 and −0.94 ​mm), and DMH (between the bregma −1.46 and −2.06 ​mm) according to the mouse brain atlas (Paxinos and Franklin, 2007). To perform a quantitative analysis, fluorescent images were obtained using a DS-Fi3 digital microscope camera (Nikon, Tokyo, Japan) under the same pinhole size, brightness, and contrast settings. Images (1440 ​× ​1024 pixels) were saved as TIF files (1440 ​× ​1024 pixels) by employing NIS Elements BR (Nikon) and arranged using Photoshop CC (Adobe Systems Incorporated, San Jose, CA). In quantitative analyses, the total area of each brain region was measured using Image J (Schneider et al., 2012). The numbers of Fos^+^ nuclei were counted using ImageJ, the threshold intensity of which was set to include measurement profiles by visual inspections and was kept constant. An analysis of all images was performed such that the experimenter was blind to the treatment group.

### 2.7 Statistics

All values are presented as the means ± s.e.m. A one-way ANOVA with repeated measures and following Tukey's post-hoc test were used to compare group differences. A difference was considered to be significant if *p* ​< ​0.05. Outlier assessment was performed by Smirnov-Grubbs test. All statistical analyses were done by using STATISTICA (StatSoft Inc., Tulsa, OK).

## 3 Results

### 3.1 Peripheral administration of low-dose LPS induced hypothermia in TRPM8 KO mice

**Fig. 1:**
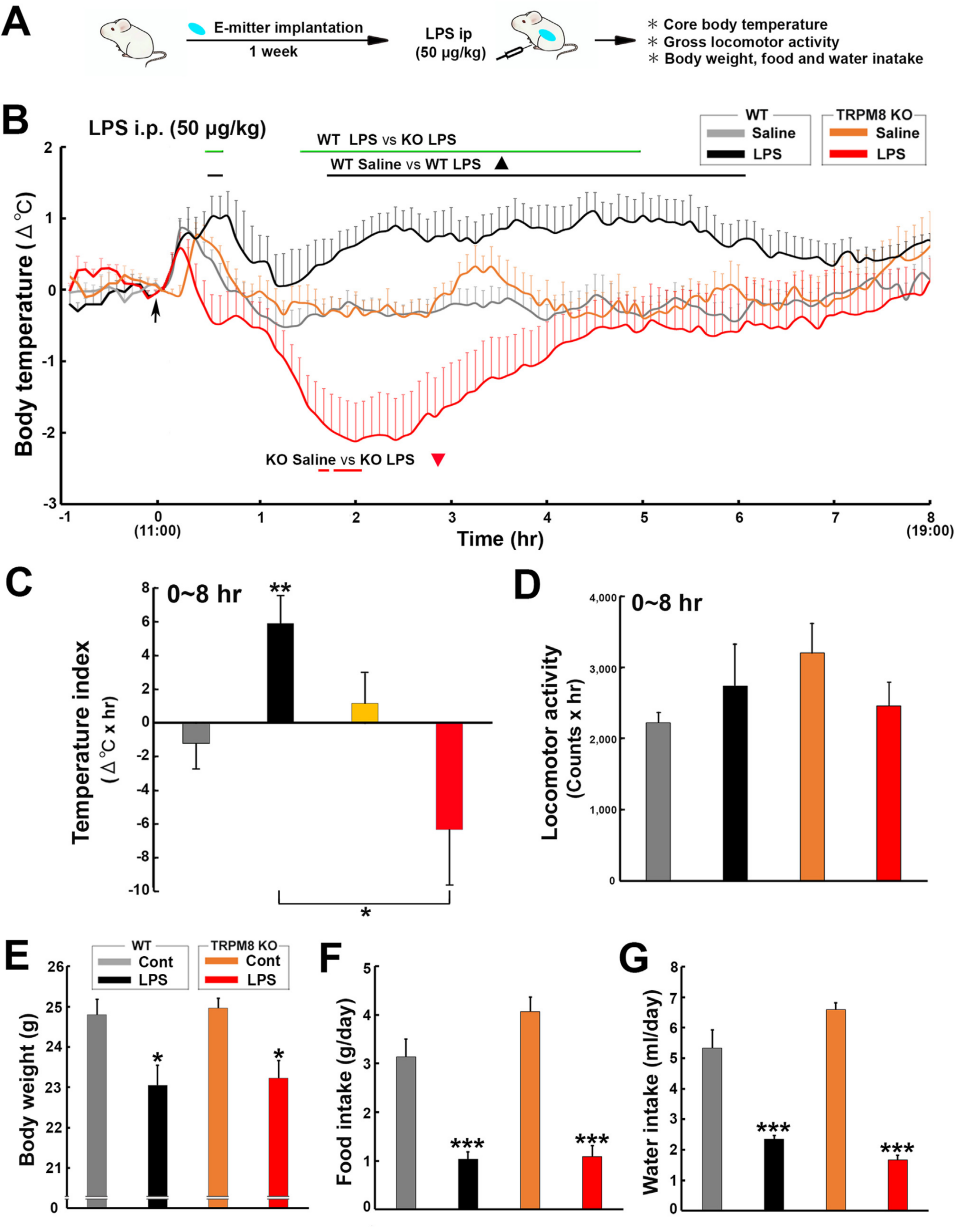
Effects of the intraperitoneal administration of a low dose of the TLR4 agonist, LPS, on the abdominal core temperature, locomotor activity, and changes in body weight, and food and water intake of WT and TRPM8 KO mice. **A**: Schematic representation showing time schedule of the experiment; the body temperature and locomotor activity of mice were measured with a G2 E-mitter transponder at an ambient temperature of 25 ​± ​0.5 ​°C after the intraperitoneal administration of 50 ​μg/kg LPS. **B**: The administration of LPS caused hypothermia in TRPM8 KO mice, whereas it induced fever in WT mice. **C**: The temperature index (Δ^o^C ​× ​hr) of LPS-treated TRPM8 KO mice was negative, whereas that of LPS-treated WT animals was positive. **D**: Cumulative locomotor activity did not significantly differ among treatment groups. **E-G**: Body weight, and food and water intake of WT and TRPM8 KO mice were significantly decreased by peripheral administration of LPS as compared with the control, but there was no significant difference between WT and TRPM8 KO animals. The control and LPS values were obtained 24 ​h before and after the administration of LPS. Data (**B-D**; WT saline, n ​= ​9; WT LPS, n ​= ​6; TRPM8 KO saline, n ​= ​5; TRPM8 KO LPS, n ​= ​11, **E-G**; n ​= ​6) are expressed as the mean (±s.e.m.). Statistically significant difference: lines in **B** indicate a significant period (*p* ​< ​0.05). ∗*p* ​< ​0.05, ∗∗*p* ​< ​0.01, ∗∗∗*p* ​< ​0.001 among treatment groups by a one-way ANOVA with Tukey's *post hoc* test.

The intraperitoneal administration of a low dose of LPS of 50–100 ​μg/kg has been shown to generate fever in mice, whereas a high dose of more than 2.5 ​mg/kg induces hypothermia (Oka et al., 2003). There was no significant difference of TLR4 expression in the CVOs and arcuate nucleus between WT and TRPM8 KO mice using the immunohistochemistry (Fig. S1). To elucidate whether TRPM8 KO mice exhibit LPS-induced fever and sickness responses, the changes of body temperature and weight, and food and water intake of WT and TRPM8 KO mice was measured after the intraperitoneal administration of low-dose (50 ​μg/kg) LPS as described in Fig. 1A. A two-way repeated measures ANOVA showed a significant effect of treatment group (F_3,243_ ​= ​20.84, *p* ​< ​0.001) and time (F_7,1944_ ​= ​4.56, *p* ​< ​0.001), but not their interaction (F_21,1940_ ​= ​0.90, *p* ​> ​0.05) on changes in core body temperature (Fig. 1B). In comparisons with mice administered saline, the body temperature of WT mice significantly (*p* ​< ​0.05) increased at 30–480 ​min and peaked (1.03 ​± ​0.35 ​°C) 40 ​min after the administration of LPS, whereas that of TRPM8 KO mice markedly decreased with a nadir (−2.12 ​± ​0.53 ​°C) at 120 ​min. There was statistically significant (*p* ​< ​0.05) difference of the body temperature between WT and TRPM8 KO mice after the administration of LPS. Mice in all treatment groups exhibited an initial stress-induced hyperthermia as a result of handling during the administration procedure, regardless of whether the endotoxin, cytokine, or saline was administered. The temperature index (Δ^o^C ​× ​hr) was significantly (*p* ​< ​0.01) higher in WT mice (5.89 ​± ​1.62) after the administration of LPS than in those administered saline (−1.18 ​± ​1.51), whereas it was significantly (*p* ​< ​0.01) lower in LPS-treated TRPM8 KO animals (−6.33 ​± ​3.26) than in those administered saline (1.15 ​± ​1.74) (Fig. 1C). The one-way ANOVA showed no significant (*p* ​> ​0.05) changes in the cumulative locomotor activity of WT and TRPM8 KO mice following the administration of LPS (Fig. 1D and Fig. S2). The one-way ANOVA showed significant (*p* ​< ​0.001) decrease in body weight, and food and water intake of WT and TRPM8 KO mice after the administration of LPS (Fig. 1E–G). The peripheral administration of low-dose LPS significantly (*p* ​< ​0.05) decreased body weight of WT and TRPM8 KO mice as compared with the control (Fig. 1E). The food and water intake were significantly (*p* ​< ​0.001) lower in LPS-treated mice than those of the control (Fig. F and G). Thus, TRPM8 KO mice developed hypothermia in contrast to fever in WT mice, in response to the peripheral administration of low-dose LPS, but there was no significant difference of sickness responses such as locomotor activity, body weight, and food and water intake between WT and TRPM8 KO mice.

### 3.2 TRPM8 KO mice developed prolonged severe hypothermia after the peripheral administration of high-dose LPS

**Fig. 2:**
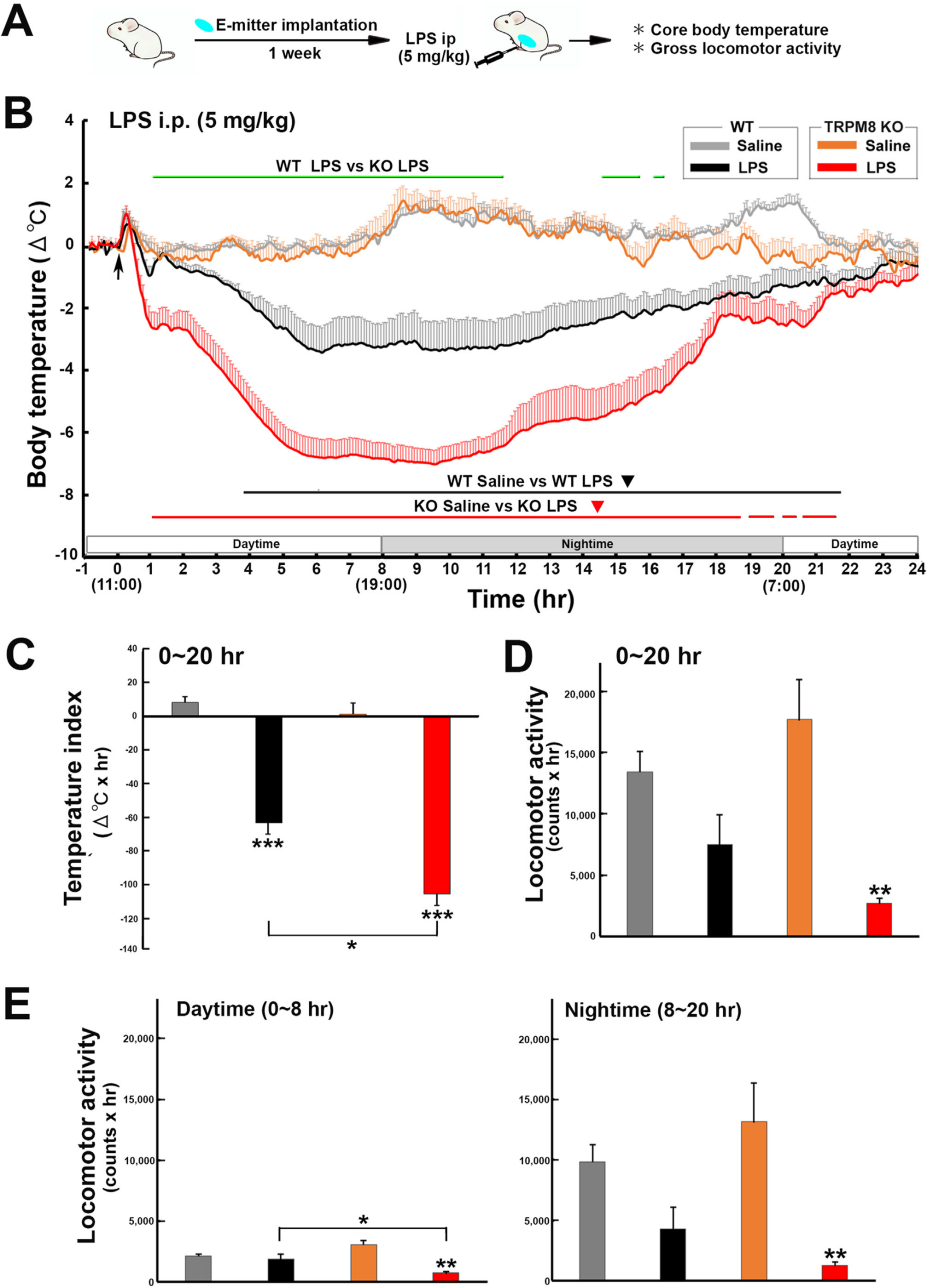
Effects of the intraperitoneal administration of a high-dose of the TLR4 agonist, LPS, on the abdominal core temperature and locomotor activity of WT and TRPM8 KO mice. **A**: Schematic illustration showing time schedule of the experiment; animals were intraperitoneally administered 5 ​mg/kg LPS and body temperature and locomotor activity were measured with a G2 E-mitter transponder at an ambient temperature of 25 ​± ​0.5 ​°C. **B**: The body temperature of WT and TRPM8 KO mice intraperitoneally administered 5 ​mg/kg LPS was significantly lower than that of mice administered saline, whereas the core body temperature of TRPM8 KO mice was significantly lower than that of WT animals following the administration of LPS. **C**: The temperature index (Δ^o^C ​× ​hr) was significantly lower in LPS-treated TRPM8 KO mice than in LPS-treated WT mice. **D,E**: The cumulative locomotor activity of LPS-treated TRPM8 KO mice during all periods examined was markedly lower than that of the saline controls, whereas that of LPS-treated WT animals did not show any significant decrease. Data (WT saline, n ​= ​9; WT LPS, n ​= ​6; TRPM8 KO saline, n ​= ​5; TRPM8 KO LPS, n ​= ​9) are expressed as the mean (±s.e.m.). Statistically significant difference: lines in **B** indicate a significant period (*p* ​< ​0.05). ∗*p* ​< ​0.05, ∗∗*p* ​< ​0.01, ∗∗∗*p* ​< ​0.001 among treatment groups by a one-way ANOVA with Tukey's *post hoc* test.

To elucidate whether TRPM8 is involved in the regulation of LPS-induced hypothermia, the body temperature of WT and TRPM8 KO mice was examined after the intraperitoneal administration of high-dose (5 ​mg/kg) LPS as described in Fig. 2A. A two-way repeated measures ANOVA revealed a significant effect of treatment group (F_3,600_ ​= ​488.27, *p* ​< ​0.001), time (F_23,13,800_ ​= ​9.33, *p* ​< ​0.001), and their interaction (F_69,13,800_ ​= ​5.59, *p* ​< ​0.001) on changes in core body temperature (Fig. 2B). Body temperature was significantly (*p* ​< ​0.05) lower in WT mice with a nadir (−3.47 ​± ​0.79 ​°C) at 365 ​min after the LPS administration than in those administered saline. Body temperature was also significantly lower in TRPM8 KO mice with a nadir (−7.04 ​± ​0.44 ​°C) at 565 ​min after the LPS administration than in those administered saline; however, LPS-induced reductions in body temperature were more prominent (*p* ​< ​0.05) in TRPM8 KO mice than in WT animals. Following the administration of LPS, the temperature index (Δ^o^C ​× ​hr) was markedly (*p* ​< ​0.001) lower in WT (−63.24 ​± ​9.43) and TRPM8 KO (−105.37 ​± ​7.01) mice than in their controls (WT, 8.17 ​± ​2.75; TRPM8 KO, 1.07 ​± ​5.96) (Fig. 2C). However, the temperature index as markedly (*p* ​< ​0.05) lower in LPS-treated TRPM8 KO mice than in WT mice. Locomotor activity during daytime and nighttime was significantly (*p* ​< ​0.01) lower in LPS-treated TRPM8 KO mice than in those administered saline (Fig. 2D,E and Fig. S3). Furthermore, the locomotor activity of LPS-treated TRPM8 KO mice during daytime was lower (*p* ​< ​0.05) than that of LPS-treated WT animals. These results indicate that TRPM8 KO mice developed severe hypothermia and exhibited reduced locomotor activity in response to the peripheral administration of high-dose LPS.

### 3.3 The central injection of LPS-induced hypothermia in TRPM8 KO mice in the early phase

**Fig. 3:**
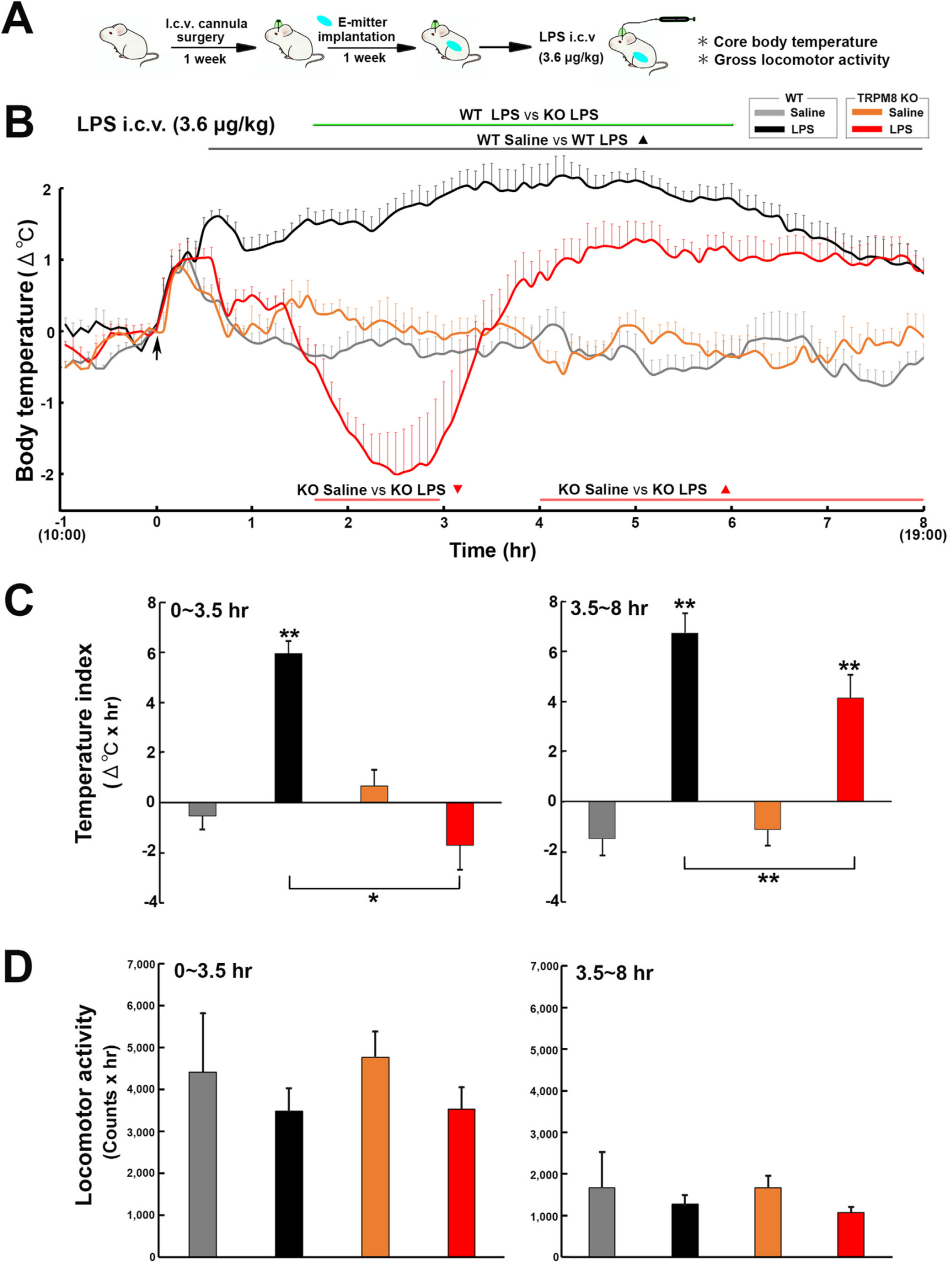
Effects of an i. c.v injection of the TLR4 agonist, LPS, on the abdominal core temperature and locomotor activity of WT and TRPM8 KO mice. **A**: Schematic diagram showing time schedule of the experiment; mice received an i.c.v. injection of 3.6 ​μg/kg LPS and body temperature and locomotor activity were measured with a G2 E-mitter transponder at an ambient temperature of 25 ​± ​0.5 ​°C. **B**: The i.c.v. injection of LPS induced prominent fever in WT mice throughout the period examined. In contrast, the LPS injection caused hypothermia in TRPM8 KO mice between 1.5 and 3.0 ​h after the injection. Body temperature in TRPM8 KO animals then gradually increased from 3.0 ​h and they developed fever after 4 ​h. **C**: The temperature index (Δ^o^C ​× ​hr) of LPS-injected TRPM8 KO mice was negative during the early phase (0–3.5 ​h), whereas that of LPS-injected WT animals was positive. The temperature index of LPS-injected WT and TRPM8 KO mice was positive in the late phase (3.5–8.0 ​h). **D**: Cumulative locomotor activity was not significantly different among treatment groups. Data (WT saline, n ​= ​6; WT LPS, n ​= ​6; TRPM8 KO saline, n ​= ​7; TRPM8 KO LPS, n ​= ​6) are expressed as the mean (±s.e.m.). Statistically significant difference: lines in **B** indicate a significant period (*p* ​< ​0.05). ∗*p* ​< ​0.05, ∗∗*p* ​< ​0.01 among treatment groups by a one-way ANOVA with Tukey's *post hoc* test.

To elucidate if central TRPM8 is involved in LPS-induced body temperature changes, the body temperature of WT and TRPM8 KO mice was measured after an i.c.v. injection of 3.6 ​μg/kg LPS as described in Fig. 3A. A two-way repeated measures ANOVA showed a significant effect of treatment group (F_3,168_ ​= ​112.04, *p* ​< ​0.001), time (F_7,1176_ ​= ​4.69, *p* ​< ​0.001), and their interaction (F_21,1176_ ​= ​7.89, *p* ​< ​0.001) on alterations in core body temperature (Fig. 3B). The body temperature of WT mice was significantly higher (*p* ​< ​0.05) at 30–480 ​min with a peak (2.19 ​± ​0.28 ​°C) 255 ​min after the i.c.v. injection of LPS than in those administered saline. In contrast to WT mice, the body temperature of TRPM8 KO mice was markedly (*p* ​< ​0.05) lower at 95–175 ​min with a nadir (−1.99 ​± ​0.59 ​°C) at 150 ​min than in those administered saline. Thereafter, it gradually returned to control levels and then was significantly (*p* ​< ​0.05) higher in the later phase than that in mice administered saline. There was statistically significant (*p* ​< ​0.05) difference of the body temperature between WT and TRPM8 KO mice after the central injection of LPS. The temperature index (Δ^o^C ​× ​hr) was significantly (*p* ​< ​0.01) higher in WT (4.94 ​± ​0.41) 0–3.5 ​h after the central LPS injection than in those administered saline (−0.45 ​± ​0.51), whereas it was significantly (*p* ​< ​0.05) lower in TRPM8 KO mice (−1.99 ​± ​0.81) than in those administered saline (0.44 ​± ​0.53) (Fig. 3C). The temperature index was significantly (*p* ​< ​0.01) higher in WT (7.59 ​± ​0.82) and TRPM8 KO (4.56 ​± ​0.99) mice 3.5–8.0 ​h after the i.c.v. injection of LPS than in those administered saline (WT, −1.52 ​± ​0.69; KO, −0.94 ​± ​0.70). The one-way ANOVA showed no significant (*p* ​> ​0.05) differences in locomotor activity in WT and TRPM8 KO mice following the i.c.v. injection of LPS (Fig. 3D and Fig. S4). These results indicate that the i.c.v. injection of LPS caused hypothermia in TRPM8 KO mice in the early phase, in contrast to fever in WT animals.

### 3.4 The peripheral administration of the TLR2 agonist zymosan induced hypothermia in TRPM8 KO mice

**Fig. 4:**
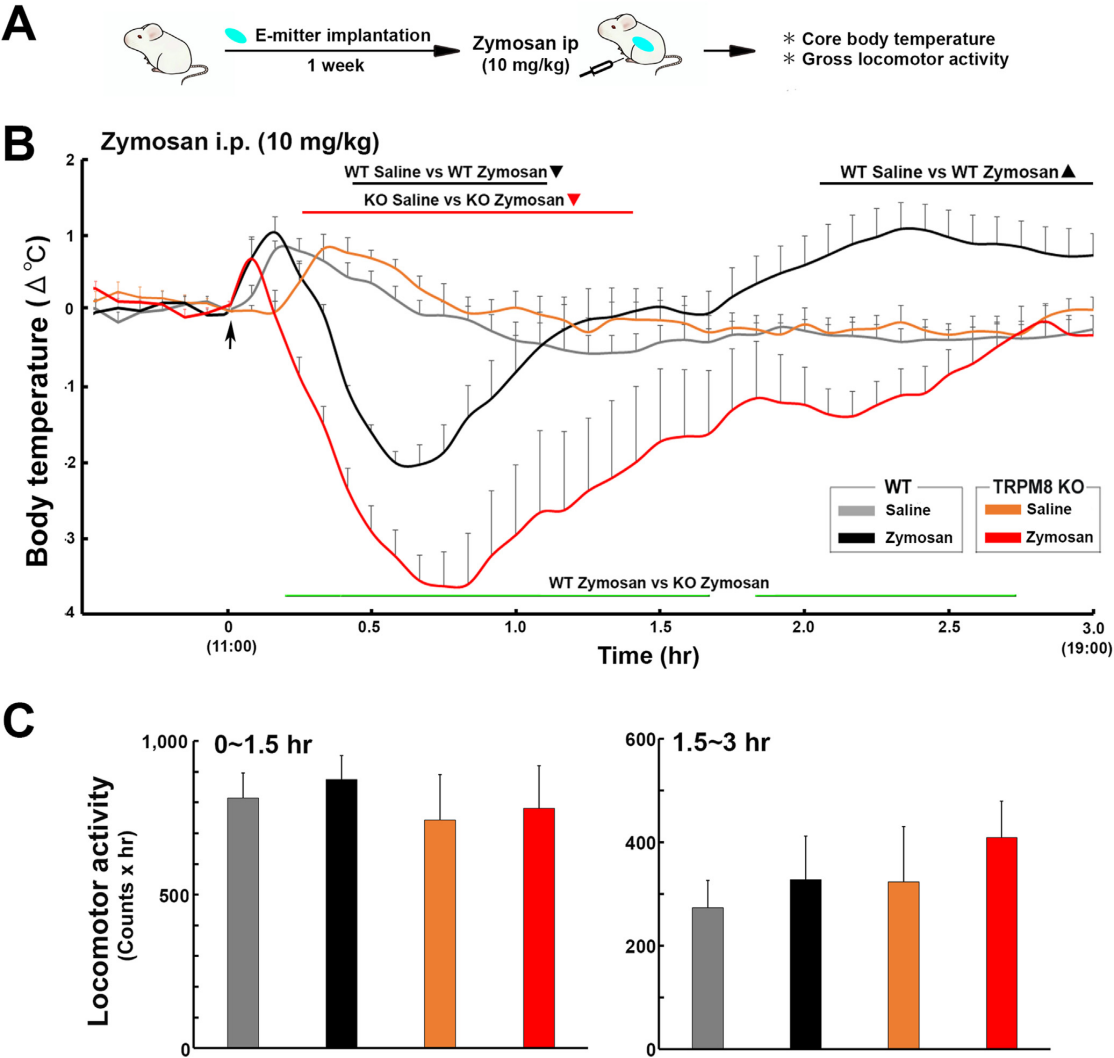
Effects of the intraperitoneal administration of the TLR2 agonist, zymosan, on the abdominal core temperature and locomotor activity of WT and TRPM8 KO mice. **A**: Schematic representation showing time schedule of the experiment; body temperature and locomotor activity were measured with a G2 E-mitter transponder at an ambient temperature of 25 ​± ​0.5 ​°C after the intraperitoneal administration of 10 ​mg/kg zymosan. **B**: The intraperitoneal administration of zymosan initially caused hypothermia and then induced fever in WT mice. However, TRPM8 KO mice exhibited hypothermia and did not show fever. **C**: Cumulative locomotor activity was not significantly different among treatment groups. Data (WT saline, n ​= ​9; WT zymosan, n ​= ​5; TRPM8 KO saline, n ​= ​5; TRPM8 KO zymosan, n ​= ​4) are expressed as the mean (±s.e.m.). Statistically significant difference: lines in **B** indicate a significant duration (*p* ​< ​0.05). ∗*p* ​< ​0.05, ∗∗*p* ​< ​0.01 among treatment groups by a one-way ANOVA with Tukey's *post hoc* test.

To elucidate whether TRPM8 is involved in TLR2-induced body temperature regulation, the body temperature of WT and TRPM8 KO mice was examined after the intraperitoneal administration of 10 ​mg/kg zymosan as described in Fig. 4A. A two-way repeated measures ANOVA showed the significant effect of treatment group (F_3,57_ ​= ​18.46, *p* ​< ​0.001), time (F_2,114_ ​= ​2.45, *p* ​> ​0.05), and their interaction (F_6,114_ ​= ​4.53, *p* ​< ​0.001) on changes in core body temperature after the intraperitoneal administration of zymosan (Fig. 4B). In comparisons with mice administered saline, WT and TRPM8 KO mice treated with zymosan initially showed transient hypothermia with a nadir at 40 (WT; −2.03 ​± ​0.26 ​°C) and 45 ​min (KO; −3.63 ​± ​0.45 ​°C). However, the extent of zymosan-induced hypothermia was more (*p* ​< ​0.05) exaggerated in TRPM8 KO mice than in the WT mice. The body temperature of WT mice then increased to generate fever with a peak (1.10 ​± ​0.34 ​°C) at 140 ​min, whereas TRPM8 KO animals did not exhibit significant (*p* ​> ​0.05) fever. The one-way ANOVA showed no significant (*p* ​> ​0.05) changes in the cumulative locomotor activity of WT and TRPM8 KO mice administered zymosan (Fig. 4C and Fig. S5). Therefore, TRPM8 KO mice only exhibited hypothermia in the early phase, in contrast to mild hypothermia in the early phase and fever in the later phase in WT animals, in response to the peripheral administration of zymosan.

### 3.5 The central injection of IL-1β may cause hypothermia in the early phase and fever in the late phase in TRPM8 KO mice

**Fig. 5:**
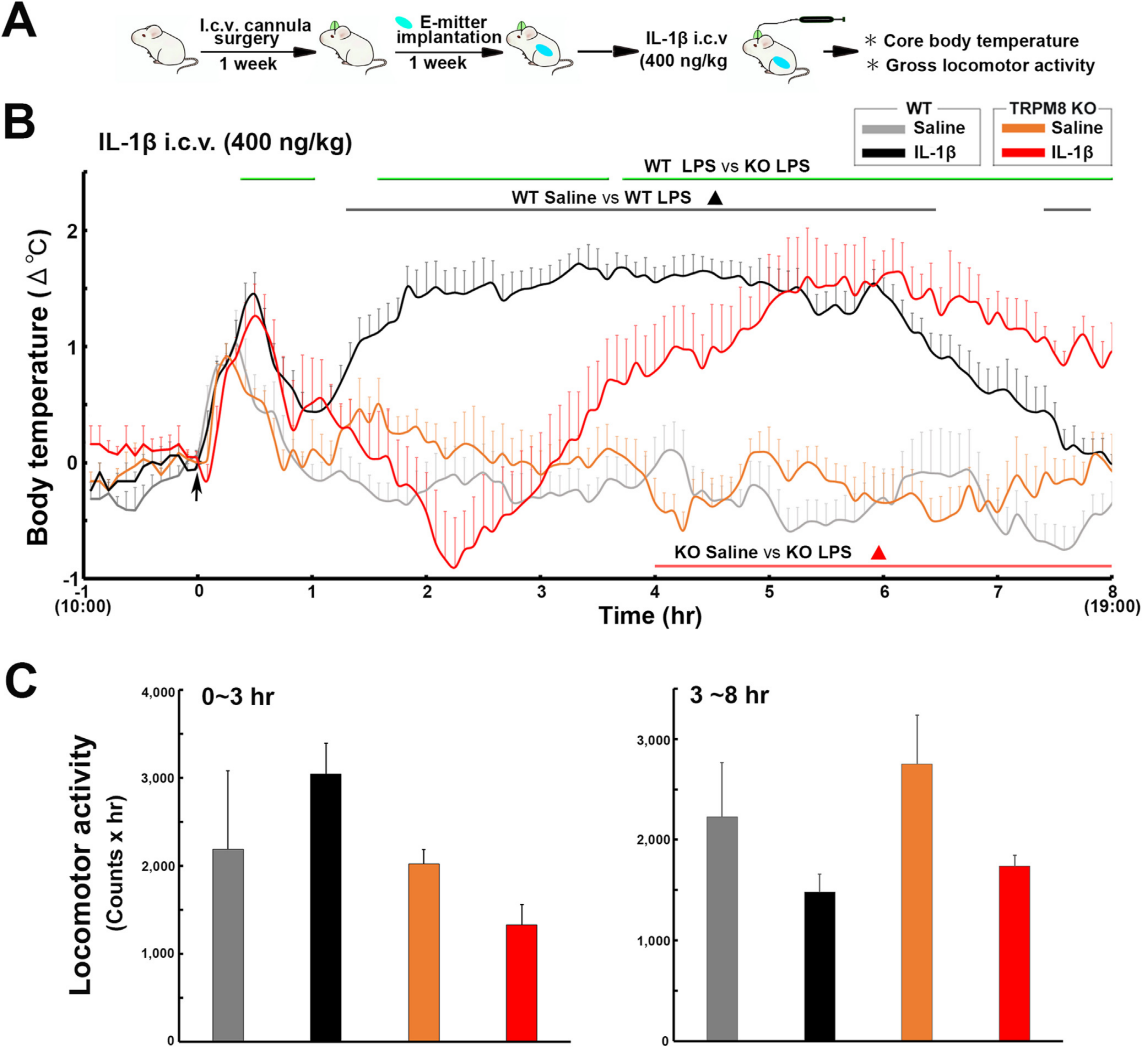
Effects of an i. c.v injection of IL-1β on the abdominal core temperature and locomotor activity of WT and TRPM8 KO mice. **A**: Schematic illustration showing time schedule of the experiment; mice received an i.c.v. injection of 400 ​ng/kg IL-1β and body temperature and locomotor activity were measured with a G2 E-mitter transponder at an ambient temperature of 25 ​± ​0.5 ​°C. **B**: The i.c.v. injection of IL-1β induced fever in WT mice, but not in their saline controls, whereas TRPM8 KO mice did not show fever in the early phase (0–3 ​h), but developed fever in the late phase (3–8 ​h). **C**: Cumulative locomotor activity was not significantly different among treatment groups. Data (WT saline, n ​= ​6; WT IL-1β, n ​= ​6; TRPM8 KO saline, n ​= ​7; TRPM8 KO Pam3CSK4, n ​= ​4) are expressed as the mean (±s. e.m.). Statistically significant difference: lines in **B** indicate a significant time (*p* ​< ​0.05). ∗∗*p* ​< ​0.01 among treatment groups by a one-way ANOVA with Tukey's *post hoc* test.

To elucidate whether central TRPM8 is involved in IL-1β-induced fever, the body temperature of WT and TRPM8 KO mice was measured after an i.c.v. injection of 400 ​ng/kg IL-1β as described in Fig. 5A. A two-way repeated measures ANOVA showed a significant effect of treatment group (F_3,152_ ​= ​89.65, *p* ​< ​0.001), time (F_7,456_ ​= ​2.54, *p* ​< ​0.05), and their interaction (F_21,456_ ​= ​5.88, *p* ​< ​0.001) on alterations in core body temperature (Fig. 5B). Body temperature was significantly (*p* ​< ​0.05) higher in WT mice at 80–205 and 440–465 ​min with a peak (1.71 ​± ​0.13 ​°C) 200 ​min after the i.c.v. IL-1β injection than in those administered saline. In contrast to WT mice, TRPM8 KO mice did not show any significant (*p* ​> ​0.05) fever within 3 ​h, but appeared to develop hypothermia. There was statistically significant (*p* ​< ​0.05) difference of the body temperature between WT and TRPM8 KO mice after the central injection of IL-1β. In comparisons with mice administered saline, the body temperature of TRPM8 KO mice gradually increased to generate significant (*p* ​< ​0.05) fever at 240–480 ​min with a peak (1.63 ​± ​0.27 ​°C) at 370 ​min. The one-way ANOVA revealed no significant (*p* ​> ​0.05) differences in locomotor activity in WT and TRPM8 KO mice following the i.c.v. IL-1β injection (Fig. 5C and Fig. S6). These results indicate that TRPM8 KO mice were more likely to develop hypothermia in the early phase and delayed fever generation in the later phase in response to the i.c.v. IL-1β injection.

### 3.6 The central injection of PGE_2_ cause fever in both WT and TRPM8 KO mice

**Fig. 6:**
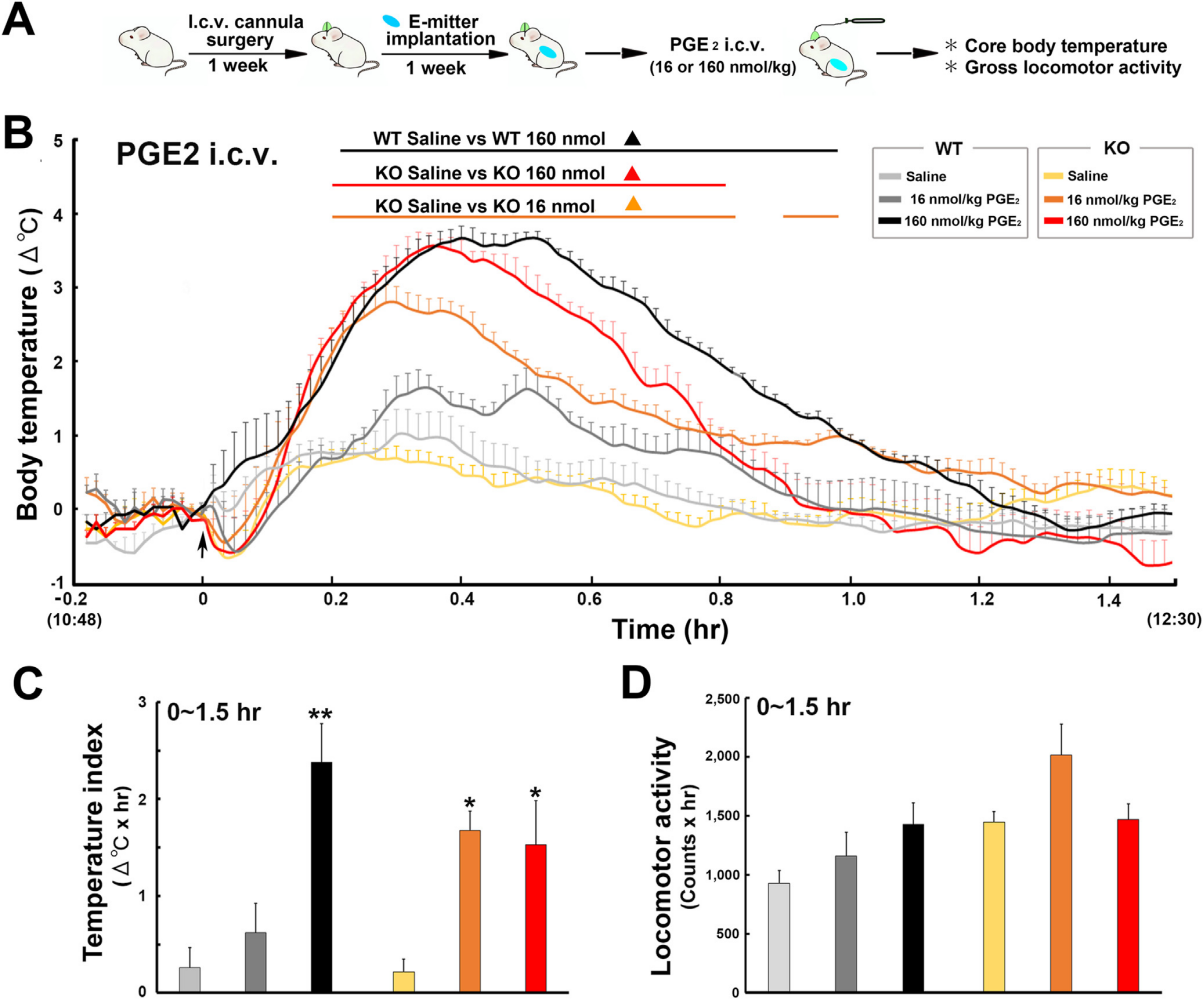
Effects of an i. c.v injection of PGE_2_ on the abdominal core temperature and locomotor activity of WT and TRPM8 KO mice. **A**: Schematic diagram showing time schedule of the experiment; mice received an i.c.v. injection of 16 or 160 ​nmol/kg PGE_2_ and body temperature and locomotor activity were measured with a G2 E-mitter transponder at an ambient temperature of 25 ​± ​0.5 ​°C. **B**: The i. c.v. injection of PGE_2_ induced prominent fever in both WT and TRPM8 KO mice. **C**: The temperature index (Δ^o^C ​× ​hr) as significantly increased in WT and TRPM8 KO mice by the i.c.v. injection of PGE_2_. **D**: Cumulative locomotor activity was not significantly different among treatment groups. Data (WT: saline, n ​= ​6; 16 ​nmol/kg PGE_2_, n ​= ​4; 160 ​nmol/kg PGE_2_, n ​= ​4; TRPM8 KO: saline, n ​= ​7; 16 ​nmol/kg PGE_2_, n ​= ​5; 160 ​nmol/kg PGE_2_) are expressed as the mean (±s.e.m.). Statistically significant difference: lines in **B** indicate a significant period (*p* ​< ​0.05). ∗*p* ​< ​0.05, ∗∗*p* ​< ​0.01 among treatment groups by a one-way ANOVA with Tukey's *post hoc* test.

To elucidate whether TRPM8 KO mice exhibit different sensitivities to PGE_2_, the body temperature of WT and TRPM8 KO mice was measured after an i.c.v. injection of PGE_2_ as described in Fig. 6A*.* A two-way repeated measures ANOVA showed a significant effect of treatment group (F_5,72_ ​= ​21.42, *p* ​< ​0.001), time (F_2,144_ ​= ​51.57, *p* ​< ​0.001), and their interaction (F_10,144_ ​= ​5.35, *p* ​< ​0.001) on changes in core body temperature (Fig. 6B). The i.c.v. injection of 160 ​nmol/kg PGE_2_ induced rapid and robust fever in both WT and TRPM8 KO mice. On the other hand, the i.c.v. injection of 16 ​nmol/kg PGE_2_ induced significant (*p* ​< ​0.05) fever in TRPM8 KO mice only. The injection of 160 ​nmol/kg PGE_2_ resulted in a significantly (*p* ​< ​0.05) higher temperature index (Δ^o^C ​× ​hr) in WT (2.38 ​± ​0.40) and TRPM8 KO (1.52 ​± ​0.45) mice than in those administered saline (WT, 0.26 ​± ​0.21; TRPM8 KO, 0.21 ​± ​0.13) (Fig. 6C). In comparisons with saline-treated mice, the temperature index was significantly (*p* ​< ​0.05) higher in TRPM8 KO mice administered 16 ​nmol/kg PGE_2_ (0.62 ​± ​0.30), but not in WT (1.67 ​± ​0.20). The temperature index was not significantly (*p* ​> ​0.05) different between WT and TRPM8 KO mice following the injection of 160 ​nmol/kg PGE_2_. Furthermore, no significant (*p* ​> ​0.05) differences were observed in locomotor activity between WT and TRPM8 KO mice following the PGE_2_ injection (Fig. 6D and Fig. S7). These results indicate that PGE_2_-dependent fever generation in the brain did not significantly differ between WT and TRPM8 KO mice.

### 3.7 Decrease of surface temperature of interscapular skin (TiScap) and back (TBack) in TRPM8 KO mice after the peripheral administration of low-dose LPS

**Fig. 7:**
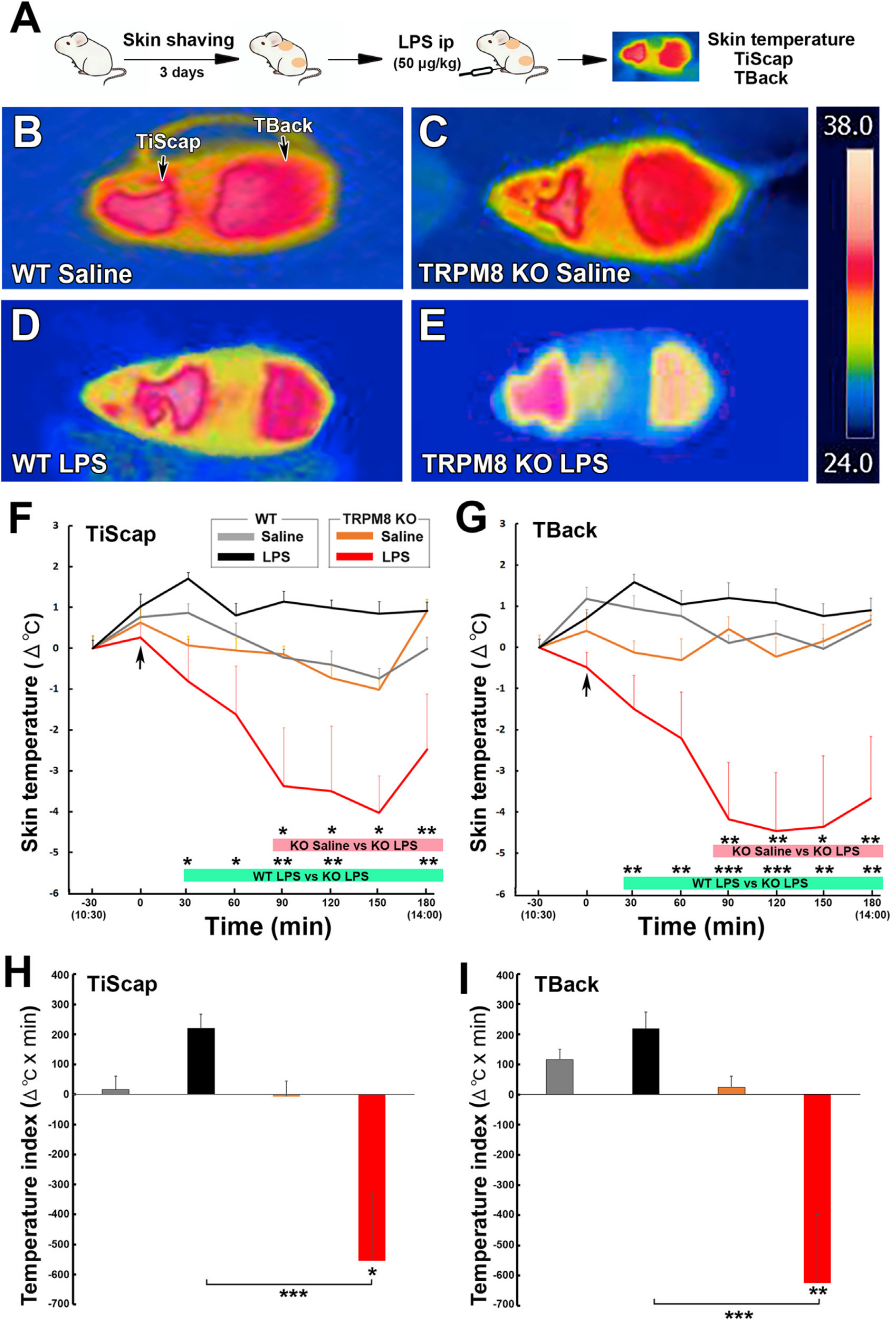
Effects of the intraperitoneal administration of a low dose of LPS on TiScap and TBack of WT and TRPM8 KO mice. **A**: Schematic representation showing time schedule of the experiment; TiScap and TBack of mice were measured with FLIR C5 infrared camera at an ambient temperature of 25 ​± ​0.5 ​°C after the intraperitoneal administration of 50 ​μg/kg LPS. **B-E**: The administration of LPS caused decline of TiScap and TBack in TRPM8 KO mice, whereas it did not change those of WT animals. Right panel showed a magnified temperature scale. **F, G**: The administration of LPS caused significant decrease of TiScap and TBack in TRPM8 KO mice, although it did not significantly alter those in WT mice. **H, I**: The temperature index (Δ^o^C ​× ​min) of TiScap and TBack in LPS-treated TRPM8 KO mice was significantly lower than that of saline-treated animals. Data (n ​= ​5) are expressed as the mean (±s.e.m.). Statistically significant difference: ∗*p* ​< ​0.05, ∗∗*p* ​< ​0.01, ∗∗∗*p* ​< ​0.001 among treatment groups by a one-way ANOVA with Tukey's *post hoc* test.

To elucidate the difference of thermogenesis in the BAT between WT and TRPM8 KO mice, TiScap and TBack was measured after the intraperitoneal administration of low-dose (50 ​μg/kg) LPS as described in Fig. 7A. TiScap and TBack are shown to be useful indicators for interscapular BAT and core body temperature, respectively (Marks et al., 2009; Vianna and Carrive, 2012). The infrared thermography showed prominent decline of TiScap and TBack in TRPM8 KO mice, in contrast to no alteration of those in WT animals (Fig. 7B–E). A two-way repeated measures ANOVA showed a significant effect of treatment group (F_3,140_ ​= ​22.98, *p* ​< ​0.001) and time (F_6,840_ ​= ​3.090, *p* ​< ​0.01), but no significant interaction between them (F_18,840_ ​= ​0.7368, *p* ​> ​0.05) on changes in TiScap (Fig. 7F). TiScap of TRPM8 KO mice significantly (*p* ​< ​0.05) decreased at 90–180 ​min and reached a nadir (−4.028 ​± ​0.903 ​°C) 150 ​min after the administration of LPS than that of saline-treated animals, whereas TiScap of WT mice was not significantly different from that of saline-treated animals. There was statistically significant (*p* ​< ​0.05) difference of TiScap between LPS-treated WT and TRPM8 KO mice.

A two-way repeated measures ANOVA showed a significant effect of treatment group (F_3,140_ ​= ​56.77, *p* ​< ​0.001) and time (F_6,840_ ​= ​2.3890, *p* ​< ​0.05), but no significant interaction between them (F_18,840_ ​= ​1.524, *p* ​> ​0.05) on changes in TBack (Fig. 7G). TBack of TRPM8 KO mice significantly (*p* ​< ​0.05) decreased at 90–180 ​min and reached a nadir (−4.458 ​± ​1.418 ​°C) 120 ​min after the administration of LPS, although that of WT mice was not significantly different from that of saline-treated animals. TBack of LPS-treated TRPM8 KO mice was statistically significant (*p* ​> ​0.05) difference from that of WT animals. The temperature index (Δ^o^C ​× ​min) of TiScap and TBack of LPS-treated TRPM8 KO mice significantly (*p* ​< ​0.001) lower than that of saline-treated TRPM8 KO and LPS-treated WT animals (Fig. 7H and I). These results indicate that TRPM8 KO mice exhibit decreased TiScap, in contrast to no changes of TiScap in WT animals in response to the peripheral administration of low-dose LPS.

### 3.8 Augmentation of fos expression in the hypothalamus in TRPM8 KO mice after the peripheral administration of low-dose LPS

**Fig. 8:**
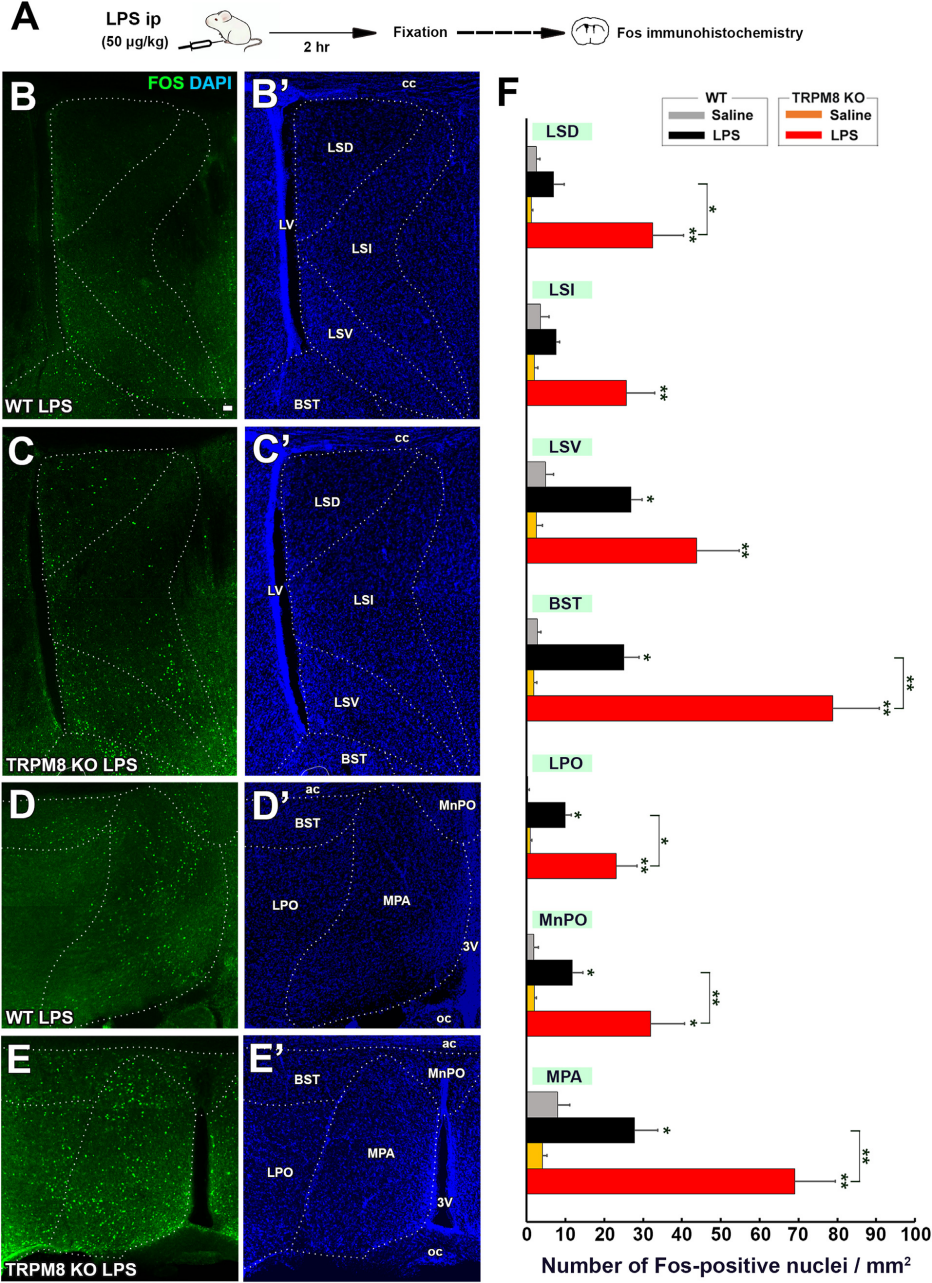
Effects of the intraperitoneal administration of a low dose of LPS on Fos expression in the POA and LS of WT and TRPM8 KO mice. **A**: Schematic representation showing time schedule of the experiment; animals were intraperitoneally administered 50 ​μg/kg LPS and were then sacrificed for Fos immunohistochemistry 2 ​h after the injection. **B-E,B-E’**: Fluorescent images revealed many Fos ​^+^ ​nuclei in the POA and LS of both WT and TRPM8 KO mice. **F**: The number of Fos^+^nuclei in the LSD, LSI, BST, and MPA was significantly higher in LPS-treated TRPM8 KO mice than in WT mice. Data (n ​= ​4) were expressed as the mean ​± ​s.e.m. Statistically significant difference: ∗: *p* ​< ​0.05, ∗∗: *p* ​< ​0.01, ∗∗∗: *p* ​< ​0.001 among treatment groups. Statistical analyses were performed using a one-way ANOVA with Tukey's post hoc test.

As shown in Fig. 1, the intraperitoneal administration of low-dose LPS induced opposite thermal responses between WT and TRPM8 KO mice: fever in WT mice and hypothermia in TRPM8 KO animals. Fos immunohistochemistry was performed to clarify the difference of hypothalamic neuronal activation between WT and TRPM8 KO mice as described in Fig. 8A. Immunohistochemistry revealed that many Fos^+^ nuclei were present in the ventral part of the LS (LSV), bed nucleus of the stria terminalis (BST), lateral preoptic area (LPO), median preoptic area (MnPO), and medial preoptic area (MPA) in LPS-treated WT mice (Fig. 8B,B′,C,C′, Figs. S8 and 9). Similarly, Fos^+^ nuclei were frequently observed in the dorsal (LSD) and intermediate part (LSI) of the LS, LSV, BST, LPO, MnPO, and MPA of LPS-treated TRPM8 KO mice (Fig. 8D,D′,E,E’, Figs. S8 and 9). The quantitative analysis showed that the number of Fos^+^ nuclei was significantly (*p* ​< ​0.05) increased in the LSV, BST, LPO, MnPO, and MPA of WT mice by the administration of LPS (Fig. 8F). In TRPM8 KO mice, the number of Fos^+^ nuclei was increased by the LPS treatment in all hypothalamic regions examined. More importantly, the number of Fos^+^ nuclei was significantly (*p* ​< ​0.05) higher in the LSD, BST, LPO, MnPO, and MPA of TRPM8 KO mice than in those of WT mice.

**Fig. 9:**
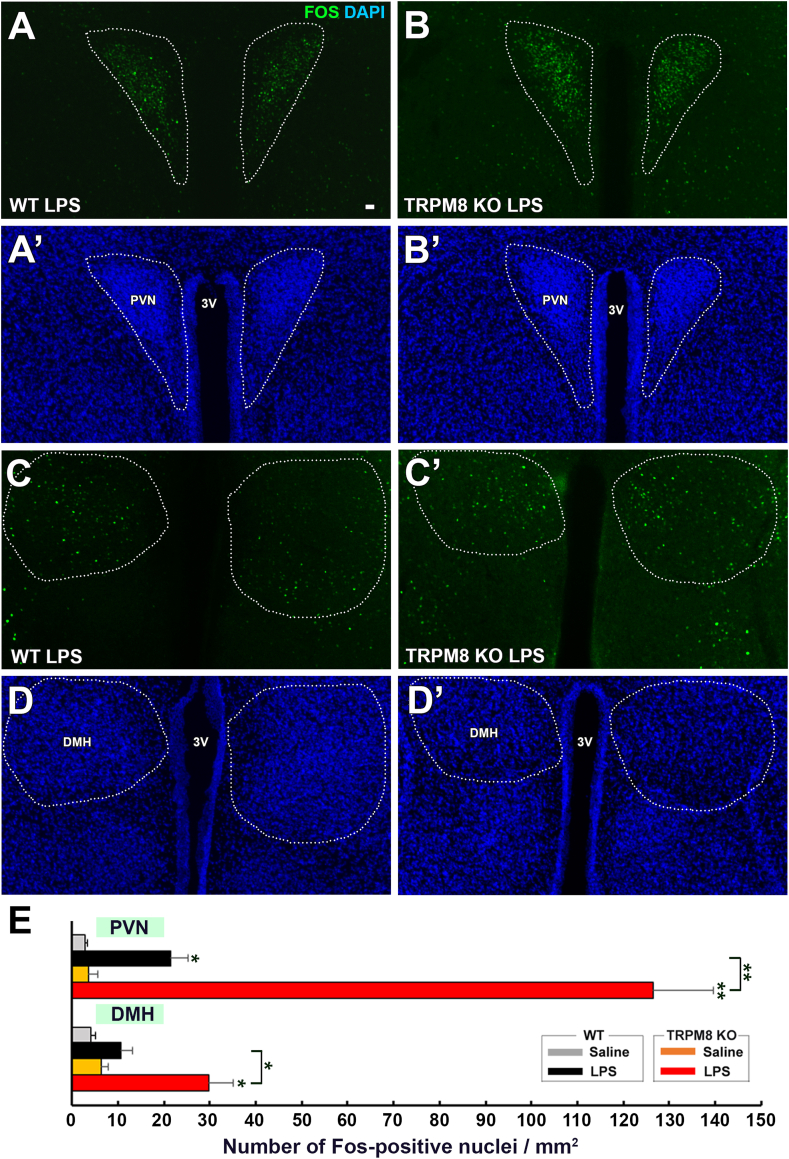
Effects of the intraperitoneal administration of a low dose of LPS on Fos expression in the PVN and DMH of WT and TRPM8 KO mouse. Animals were intraperitoneally administered 50 ​μg/kg LPS and were then sacrificed for Fos immunohistochemistry 2 ​h after the injection. **A-D,A-D’**: Fluorescent images revealed many Fos ​^+^ ​nuclei in the PVN and DMH of both WT and TRPM8 KO mice. **E**: The number of Fos^+^nuclei in the PVN and DMH was significantly higher in LPS-treated TRPM8 KO mice than in WT mice. Data (n ​= ​4) were expressed as the mean ​± ​s.e.m. Statistically significant difference: ∗: *p ​<* 0.05, ∗∗: *p* ​< ​0.01, ∗∗∗: *p* ​< ​0.001 among treatment groups by a one-way ANOVA with Tukey's post hoc test.

The number of Fos^+^ nuclei in the PVN and DMH was higher in LPS-treated WT mice (Fig. 9A,A′,C,C’) than in saline-treated mice (Fig. S10). Fos^+^ nuclei were more frequently observed in the PVN and DMH of LPS-treated TRPM8 KO mice than in WT mice. The number of Fos^+^ nuclei was significantly (*p* ​< ​0.05) increased in the PVN and DMH of WT and TRPM8 KO animals by the administration of LPS, but was significantly (*p* ​< ​0.05) higher in LPS-treated TRPM8 KO mice than in WT mice (Fig. 9C). These results indicate that Fos expression in the hypothalamus was stronger in TRPM8 KO mice than in WT animals following the peripheral administration of low-dose LPS.

## 4 Discussion

TRPM8 functions as a neuronal sensor of cold temperatures and is activated by moderate cooling and cooling agents, such as menthol and icilin (McKemy et al., 2002). TRPM8-deficient mice show no preference for warm temperatures over cold temperatures and have impaired cold avoidance behavior (Bautista et al., 2007; Dhaka et al., 2007). Besides its role in cold sensation, TRPM8 is also involved in the suppression of inflammatory responses in colitis (Ramachandran et al., 2013), experimental autoimmune encephalomyelitis (Ewanchuk et al., 2018), and the augmentation of airway inflammatory diseases (Liu et al., 2018). However, no information is currently available on the functional significance of TRPM8 in endotoxin-induced body temperature alterations. In the present study, we found that the peripheral administration of low-dose LPS induced hypothermia in TRPM8 KO mice, in contrast to fever in WT animals. Moreover, hypothermia was more severe in TRPM8 KO mice than in WT mice after the peripheral administration of high-dose LPS. Similar to TLR4 signaling, the peripheral administration of the TLR2 agonist zymosan only induced hypothermia in TRPM8 KO mice, in contrast to mild hypothermia in the early phase and fever in the later phase in WT animals. Therefore, the present study is the first to show that TRPM8 is important for switching between fever and hypothermia under endotoxin-induced inflammation.

The present study revealed that the i.c.v. LPS injection induced hypothermia in TRPM8 KO mice contrary to fever in WT animals, as the intraperitoneal administration of low-dose (50 ​μg/kg) LPS. The i.c.v. injections of LPS, which is sufficient to cause hypothermia by peripheral administration, induces only fever (Liu et al., 2012; Al-Saffar et al., 2013). LPS receptor, TLR4, is expressed at astrocytes/tanycytes of the adult mouse CVOs (Chakravarty and Herkenham, 2005; Nakano et al., 2015) and peripheral and central administration of LPS directly activates TLR4 to induce inflammatory signaling at TLR4-expressing astrocytes/tanycytes of the CVOs (Nakano et al., 2015; Yoshida et al., 2016; Vargas-Caraveo et al., 2017; Muneoka et al., 2019). Moreover, i.c.v. LPS injection causes Fos expression in the hypothalamus and brainstem A2 neurons, and increases plasma corticosterone levels and heart rare (Wan et al., 1993; Xia and Krukoff, 2003). LPS at the concentration of 100 ​μg/ml directly stimulates neurons and astrocytes of the organum vasculosum of the lamina terminalis (Ott et al., 2010) and TRPM8-transfectted HEK293T cells (Boonen et al., 2018) to cause calcium ion influx *in vitro*, but we can deny the both possibilities because we used the i.c.v. injection of LPS at the concentration of 30 ​μg/ml and LPS was diluted to 2.3 ​μg/ml by estimating CSF volume 36 ​μl for adult mouse (Rudick et al., 1982). Thus, centrally injected LPS directly activates TLR4 of the brain and then generates fever in WT mice and hypothermia in TRPM8 KO animals. Furthermore, the present data showed that the i.c.v. injection of IL-1β tended to cause hypothermia and did not induce fever. The peripheral and central administration of IL-1β causes fever, but it never induces hypothermia (Klir et al., 1994; Luheshi et al., 1996). Thus, the present study indicates that TRPM8 is necessary for intrinsic properties of brain circuits to generate proper body temperature alteration in response to peripheral LPS or zymosan administration or central LPS or IL-1β injection.

Although central injection of both LPS and IL-1β elicited hypothermia in TRPM8 KO mouse, their body temperature decrease was seen only in the early phase and then it returned to control levels in the later phase. In IL-6 KO mice, the peripheral administration of low-dose (50 ​μg/kg) LPS and central injection of IL-1β could not elicits fever, but that of high-dose (2.5 ​mg/kg) LPS induces hypothermia (Chai et al., 1996; Kozak et al., 1998; Leon, 2004). In IL-1β KO mice, however, peripheral administration of low-dose (100 ​μg/kg) and high-dose (2.5 ​mg/kg) LPS normally elicits fever and hypothermia, respectively (Kozak et al., 1998; Leon, 2004). The peripheral and central injection of TNF-α induces hypothermia by decreasing sympathetic nerve activity controlling the BAT (Holt et al., 1989; Kozak et al., 1995). The i. c.v. injection of LPS induces Fos expression in the PVN and A2 region of the solitary nucleus, but intraperitoneal administration of LPS induces Fos expression in more broad hypothalamic and brainstem regions (Wan et al., 1993). Taken together, the present study suggests that i.c.v. injection of LPS and IL-1β mimics brain conditions with peripherally stimulated animals in the early phase, but it is not maintained in the later phase possibly due to the differences of cytokine production and/or activation of neural circuits.

PGE_2_ is the ultimate mediator of fever response by acting on the POA of the hypothalamus (Scammell et al., 1996; Lazarus et al., 2007). The present study showed that the i.c.v. injection of PGE_2_ generated fever in TRPM8 KO mice in a similar manner to WT mice, indicating that TRPM8 KO mice preserve the ability generating fever. The i.c.v. injection of PGE_2_ causes Fos expression in many brain regions that are similar to that induced by peripheral endotoxin challenge (Lacroix et al., 1996). PGE_2_ and cytokine levels in plasma and POA are higher in rats exhibiting hypothermia than in fever-generating animals (Saramago et al., 2019). Fos expression in the PVN and DMH was previously reported to be stronger in mice with hypothermia than in fever-generating animals after the administration of LPS (Wanner et al., 2013; Mota et al., 2019). In the present study, the number of Fos^+^ nuclei was higher in the POA, LS, PVN, and DMH of TRPM8 KO mice than in WT animals after the peripheral administration of low-dose LPS. The activation of opsin 5- or pyroglutamylated RFamide peptide-expressing neurons in the POA acts on the DMH and then induces hypothermia together with decreases in the metabolic rate (Hrvatin et al., 2020; Takahashi et al., 2020; Zhang et al., 2020). In contrast, the activation of bombesin-like receptor 3-expressing neurons in the POA causes fever and heart (Piñol et al., 2021). Previous findings showing that TRPM8^+^ somata are localized at the medial and lateral POA and lateral septum and that of axonal fibers are seen at POA and DMH supports this possibility (Ordas et al., 2019). The present study showed that sickness responses such as decrease in locomotor activity, body weight, and food and water intake were not different between WT and TRPM8 KO mice with the administration of low-dose (50 ​μg/kg) LPS. However, hypothermic responses were more severe in TRPM8 KO mice than in WT mice following the peripheral administration of high-dose (5 ​mg/kg) LPS. Furthermore, locomotor activity was significantly lower in TRPM8 KO mice than in WT mice after the administration of high-dose (5 ​mg/kg) LPS. A principal mechanism of hypothermia in the endotoxin-induced inflammation is considered to be decrease of metabolic heat production (Romanovsky et al., 2005; Garami et al., 2018). The present study revealed that TiScap in TRPM8 KO mice was prominently decreased with the peripheral administration of low-dose (50 ​μg/kg) LPS. The activity of vascular constriction of skin surface, thermogenesis of the BAT, and shivering in skeletal muscle is regulated by the hypothalamic thermoregulatory neurons (Romanovsky et al., 1996, 2005, 2007; Garami et al., 2018). Thus, the present results indicate that TRPM8 participates in hypothalamic circuits for proper generation of fever and hypothermia, or switching between fever and hypothermia.

The peripheral, but not central, administration of high-dose TRPM8 antagonist induces hypothermia under subneutral ambient temperature, while that of low-dose antagonist causes hypothermia upon exposure to low ambient temperature (Knowlton et al., 2011; Almeida et al., 2012). These results suggest that peripheral TRPM8 is consecutively activated to maintain normal body temperature, but central TRPM8 is inactive state under subneutral conditions. Moreover, greater heat dissipation is reported in TRPM8 KO and TRPM8 antagonist-treated animals possibly due to increased tail skin surface vasodilation (Almeida et al., 2012; Reimúndez et al., 2018). However, recently, tailless mice show similar body temperature, metabolic rates, and heat conductances to control animals and heat dissipation, indicating that the tail contributes only 5–8% of whole body in thermoregulation (Škop et al., 2020). TRPM8 KO mice develop late-onset obesity and metabolic dysfunction under mild-cold (21 ​°C) ambient temperature (Reimúndez et al., 2018). The vasodilation of skin vessels is quite sensitive to ambient temperature, it is rarely observed under ambient temperature beneath 29/30 ​°C known as thermoneutral for mice (Wanner et al., 2017; Garami et al., 2018). The peripheral administration of fever-generating or low-dose (60 ​μg/kg) LPS increases sympathetic nervous activity via resetting of the baroflex neural arc and does not induce hypotension (Tohyama et al., 2018), whereas that of hypothermia-generating or high-dose LPS (20 ​mg/kg) causes hypotension (Vayssettes-Courchay et al., 2005). In the present study, we found that the peripheral administration of low-dose (50 ​μg/kg) LPS prominently decreased TiScap, an indicator of BAT temperature, in TRPM8 KO mice, but it did not change TiScap in WT animals. TRPM8 KO mice showed normal fever generation by the BAT with β3-adrenergic receptor activation (Reimúndez et al., 2018). The thermoregulatory responses such as vascular constriction or dilation of skin surface, thermogenesis of the BAT, liver, brain, and skeletal muscle is coordinately controlled by the autonomic nervous system (Romanovsky et al., 1996, 2005, 2007; Garami et al., 2018). Taken together with the previous reports, it is suggested that TRPM8 governs not only the brain circuits to control autonomic nervous system for thermogenesis but also vascular response of skin surface in peripheral tissues.

## 5 Conclusion

In conclusion, thus the present study provides evidence that TRPM8 has crucial function for switching between fever and hypothermia during endotoxin-induced inflammation. The peripheral administration of low-dose LPS and zymosan caused hypothermia in TRPM8 KO mice in contrast to fever in WT animals. Moreover, TRPM8 KO mice showed severe hypothermia after the peripheral administration of high-dose LPS. A central injection of LPS or Il-1β also generated hypothermia in TRPM8 KO mice. Infrared thermography revealed prominent decline of the interscapular skin temperature compatible to the BAT one. Fos immunohistochemistry revealed intense Fos activation of hypothalamic thermoregulation-associated nuclei in TRPM8 KO mice after the peripheral administration of low-dose LPS.

## Declaration of competing interest

The authors declare that there is no conﬂict of interest that may be perceived as prejudicing the impartiality of the research reported.
