# Supplementary material for: Role of TRPM8 in switching between fever and hypothermia in adult mice during endotoxin-induced inflammation
Source: Brain Behav Immun Health. 2021 Jun 30;16:100291. doi: 10.1016/j.bbih.2021.100291 (PMC8474285; doi:10.1016/j.bbih.2021.100291)
Supplement: Multimedia component 1 [file mmc1.pdf]

Brain, Behavior, and Immunity - Health

<u>Supplementary Figures</u>

Role of TRPM8 in switching between fever and hypothermia in adult mice during endotoxin-induced inflammation

Chinatsu Shiraki*, Ririka Horikawa*, Yuzuki Oe, Momoka Fujimoto, Kaho Okamoto, Erkin Kurganov, Seiji Miyata

Department of Applied Biology, Kyoto Institute of Technology, Matsugasaki, Sakyo-ku, Kyoto 606-8585, Japan.

Correspondence:

Seiji Miyata (smiyata@kit.ac.jp)
Department of Applied Biology, Kyoto Institute of Technology, Kyoto 606-8585, Japan.

*Equal contribution

Supplementary Figure 1

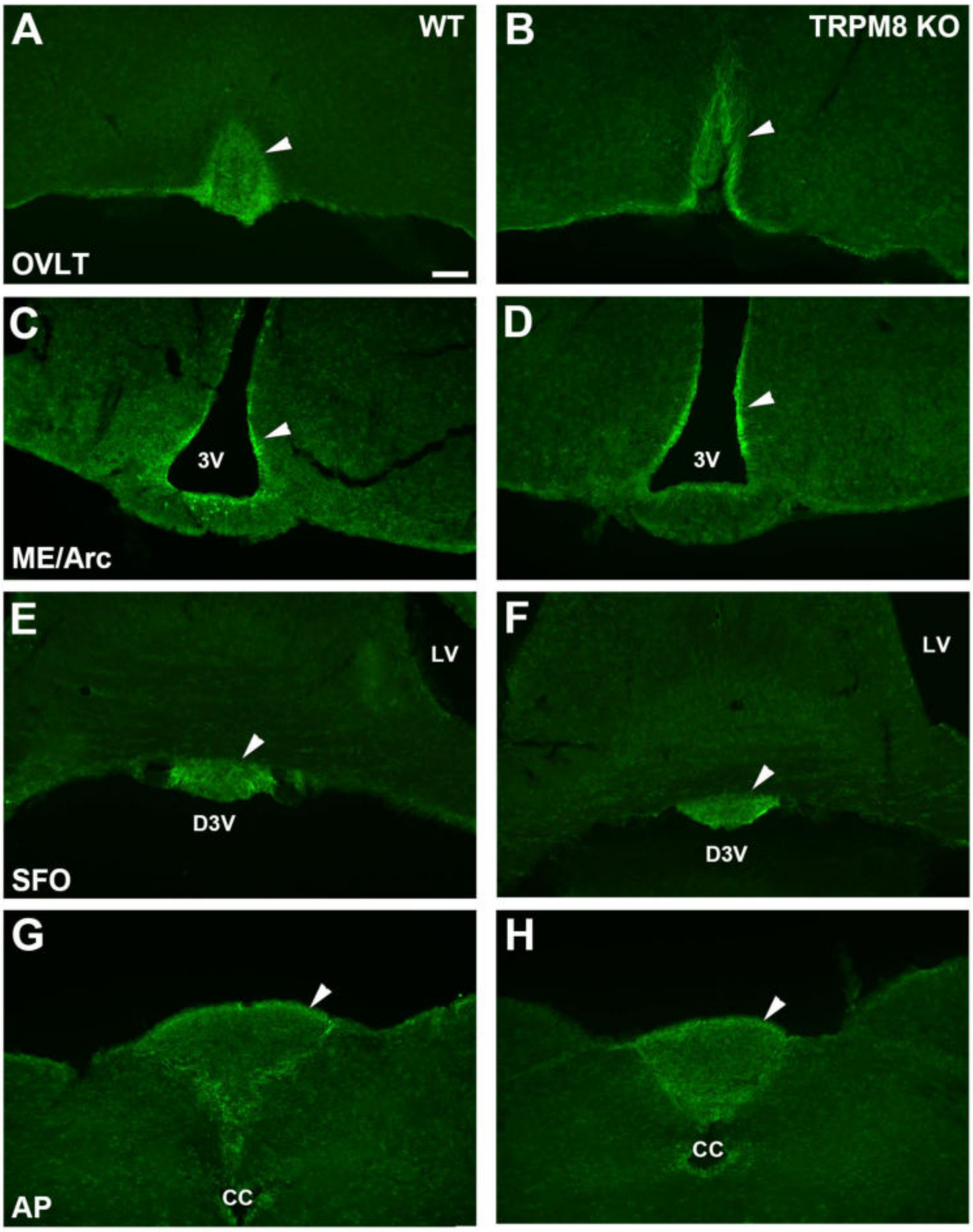

Fig. S1 Expression of TLR4 in the CVOs and median eminence/arcuate nucleus of WT and TRPM8 KO mice. The immunohistochemistry showed that TLR4 expression (arrowheads) in the CVOs and median eminence/arcuate nucleus of WT mice (A,C,E,G) was same to that of TRPM8 KO animals (B,D,F,H). 3V, 3rd lateral ventricle; D3V, dorsal 3rd ventricle; CC, central canal. Scale bar = 100 μm.

Supplementary Figure 2

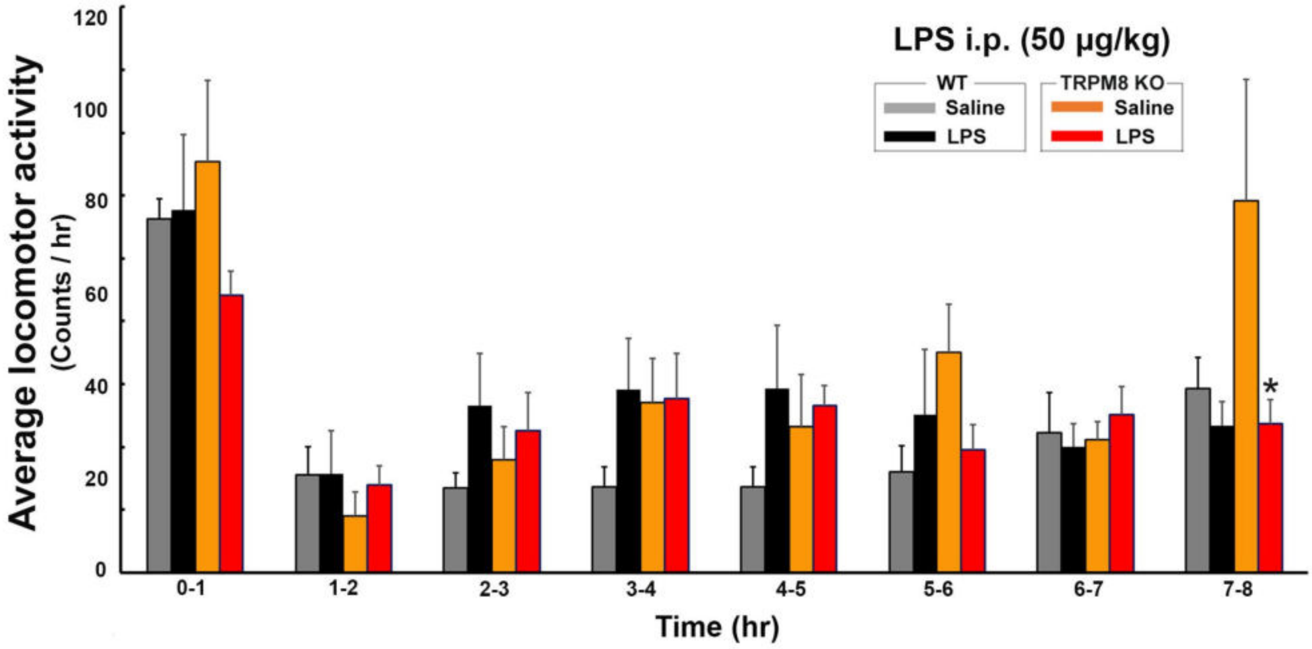

Fig. S2 Effects of the intraperitoneal administration of 50 μg/kg LPS on the locomotor activity of WT and TRPM8 KO mice. No significant differences were observed between saline- and LPS-treated WT and TRPM8 KO mice. Data (WT saline, n = 9; WT LPS, n = 6; TRPM8 KO saline, n = 5; TRPM8 KO LPS, n = 11) are expressed as the mean (± s.e.m.). * $p < 0.05$ vs saline by a one-way ANOVA with Tukey's *post hoc* test.

Supplementary Figure 3

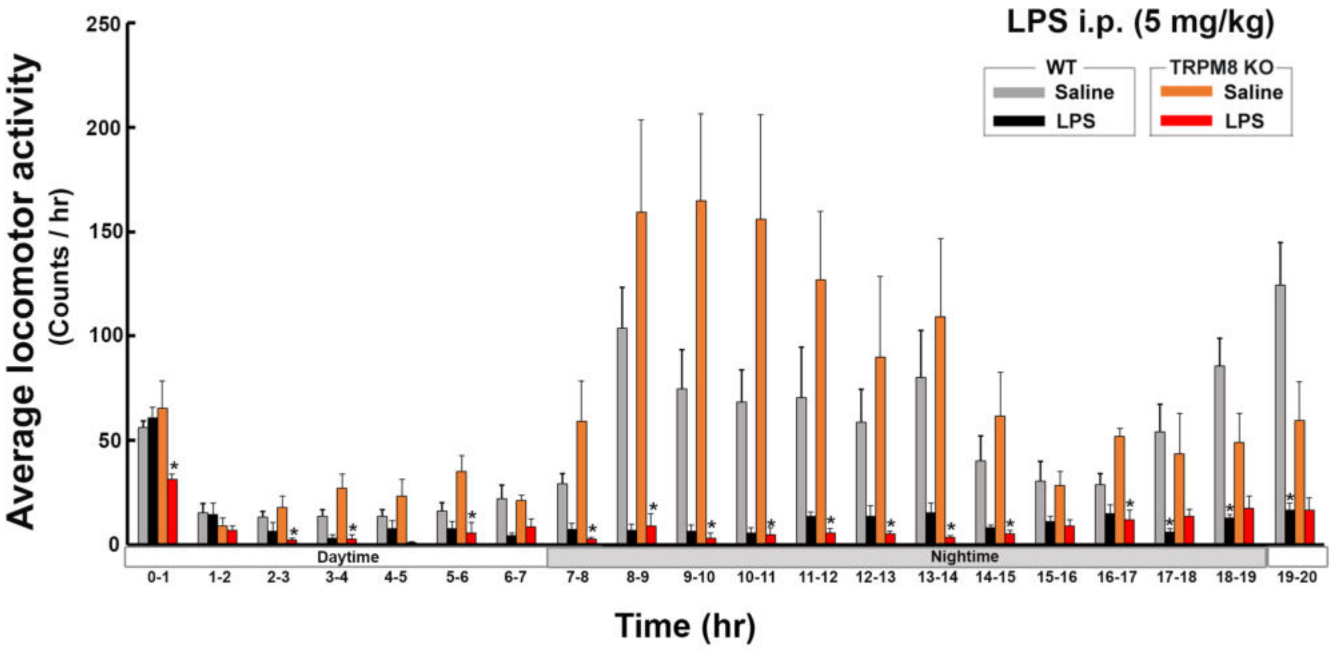

Fig. S3 Effects of the intraperitoneal administration of 5 mg/kg LPS on the locomotor activity of WT and TRPM8 KO mice. The locomotor activity of LPS-treated TRPM8 KO mice was often lower than that of the saline control, whereas LPS-treated WT animals sometimes showed reduced locomotor activity. Data (WT saline, n = 9; WT LPS, n = 6; TRPM8 KO saline, n = 5; TRPM8 KO LPS, n = 9) are expressed as the mean (± s.e.m.). * $p < 0.05$ vs saline by a one-way ANOVA with Tukey's *post hoc* test.

Supplementary Figure 4

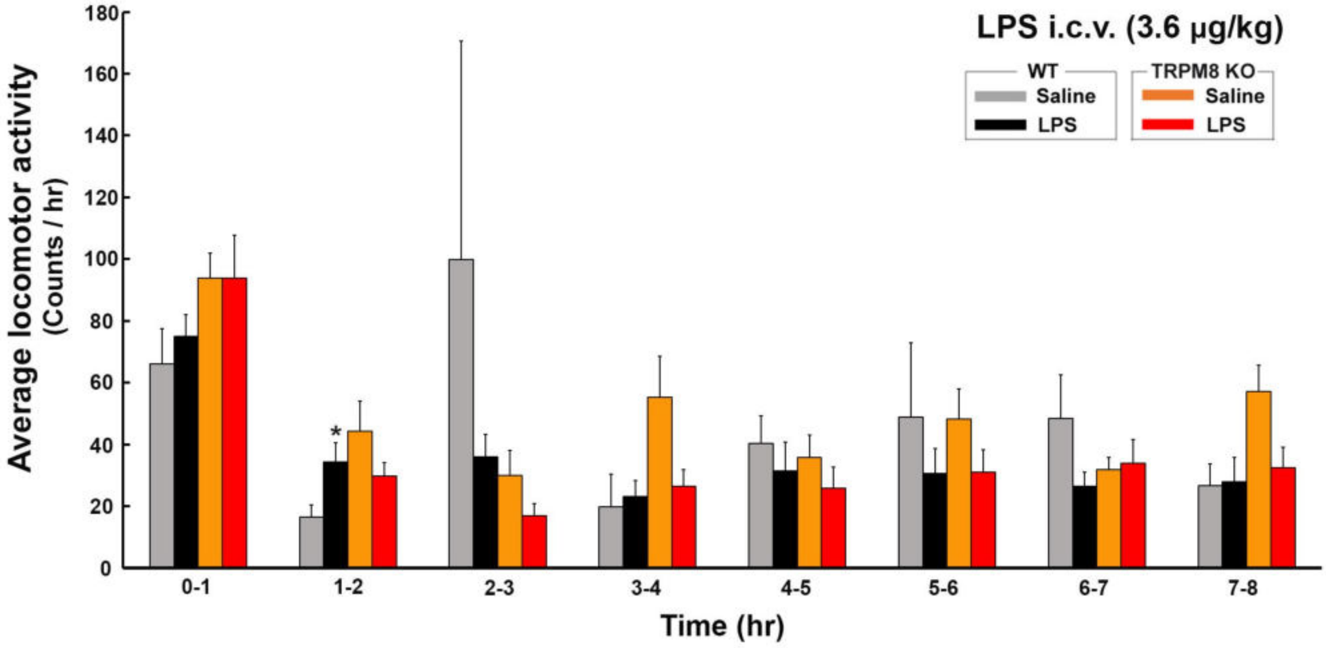

Fig. S4 Effects of an i.c.v injection of 3.6 μg/kg LPS on the locomotor activity of WT and TRPM8 KO mice. No significant difference was observed between saline- and LPS-injected WT and TRPM8 KO mice. Data (WT saline, n = 6; WT LPS, n = 6; TRPM8 KO saline, n = 7; TRPM8 KO LPS, n = 6) are expressed as the mean (± s.e.m.). * $p < 0.05$ vs saline by a one-way ANOVA with Tukey's *post hoc* test.

Supplementary Figure 5

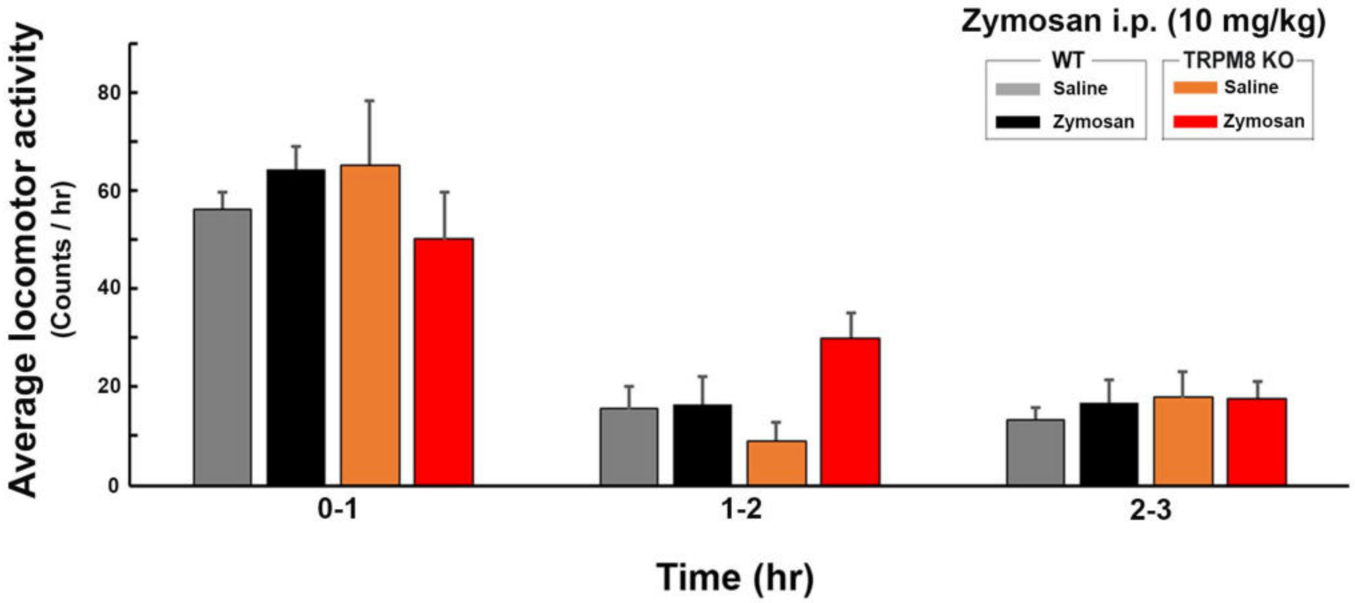

Fig. S5 Effects of the intraperitoneal administration of 10 mg/kg zymosan on the locomotor activity of WT and TRPM8 KO mice. No significant difference was observed between saline- and zymosan-treated WT and TRPM8 KO mice. Data (WT saline, n = 9; WT zymosan, n = 5; TRPM8 KO saline, n = 5; TRPM8 KO zymosan, n = 4) are expressed as the mean (± s.e.m.). Statistical analyses were performed by a one-way ANOVA with Tukey's *post hoc* test.

Supplementary Figure 6

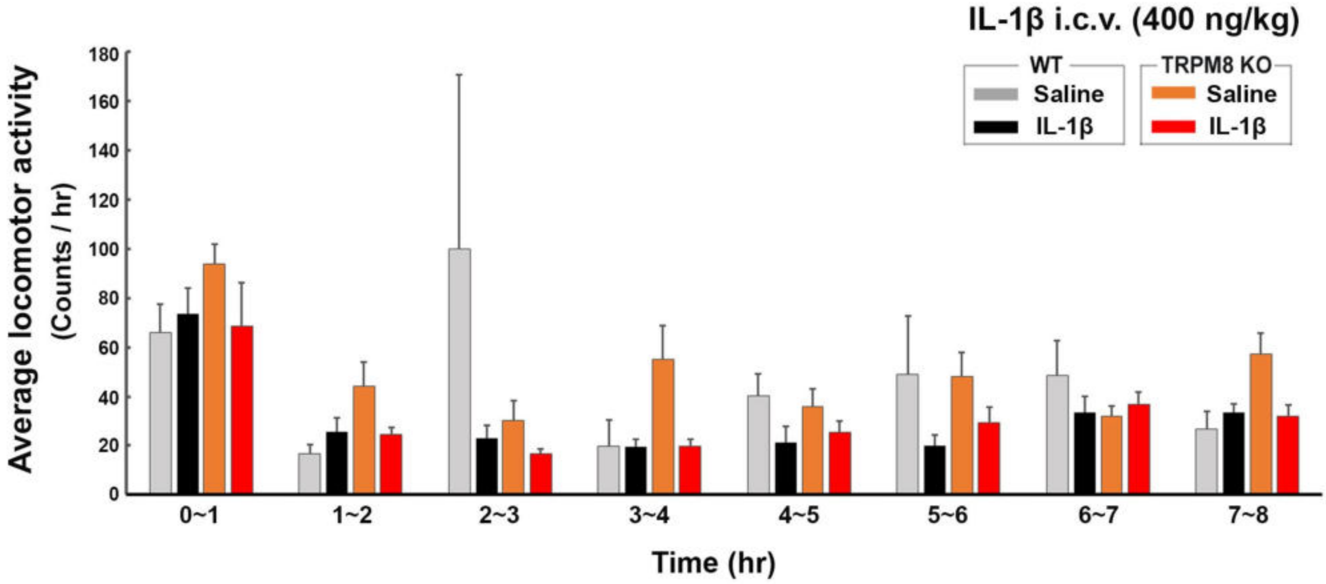

Fig. S6 Effects of an i.c.v injection of 400 ng/kg IL-1β on abdominal core temperatures and locomotor activities of WT and TRPM8 KO mice. No significant difference was detected between saline- and IL-1β-injected WT and TRPM8 KO mice. Data (WT saline, n = 6; WT IL-1β, n = 6; TRPM8 KO saline, n = 7; TRPM8 KO Pam3CSK4, n = 4) are expressed as the mean (± s.e.m.). Statistical analyses were performed by a one-way ANOVA with Tukey's *post hoc* test.

Supplementary Figure 7

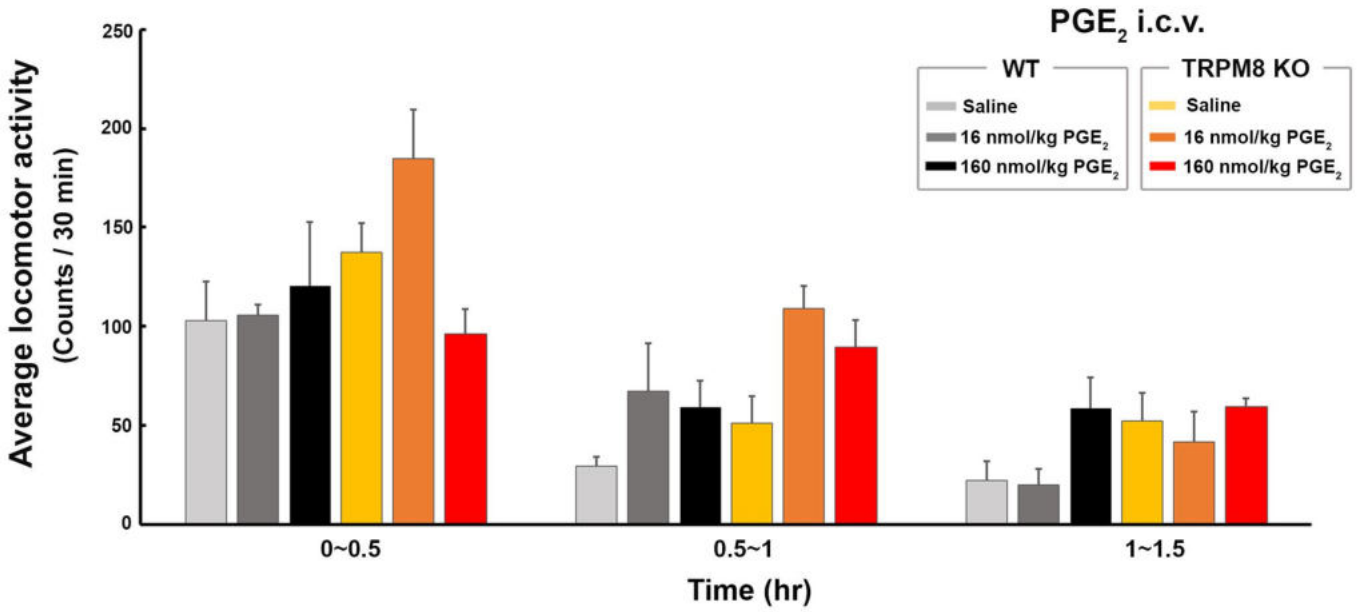

Fig. S7 Effects of an i.c.v. injection of 16 and 160 nmol/kg PGE_2 on the locomotor activity of WT and TRPM8 KO mice. No significant difference was observed between saline- and PGE_2-injected WT and TRPM8 KO mice. Data (WT: saline, n = 6; 16 nmol/kg PGE_2, n = 4; 160 nmol/kg PGE_2, n = 4; TRPM8 KO: saline, n = 7; 16 nmol/kg PGE_2, n = 5; 160 nmol/kg PGE_2) are expressed as the mean (± s.e.m.). Statistical analyses were performed by a one-way ANOVA with Tukey's post hoc test.

Supplementary Figure 8

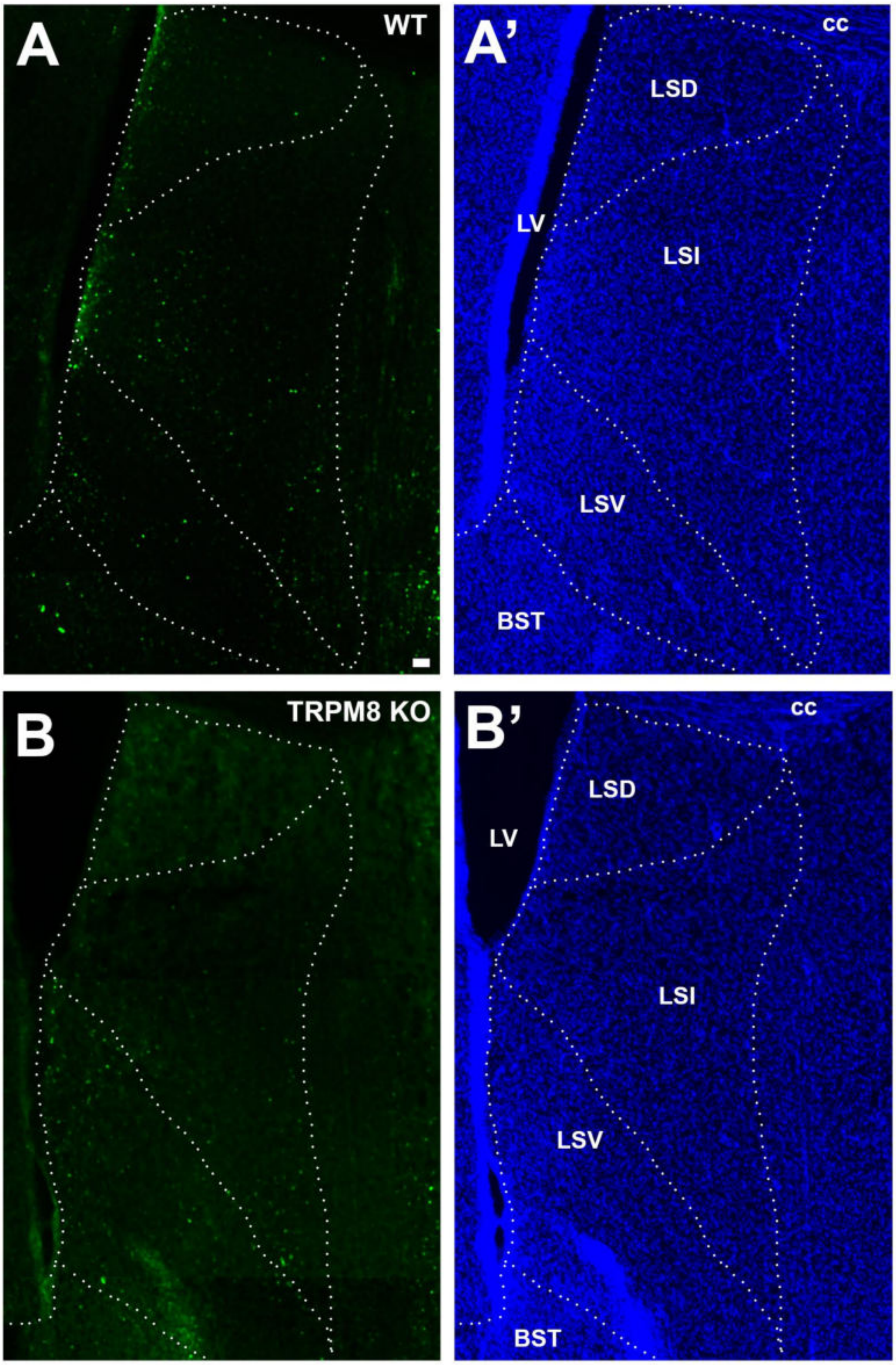

Fig. S8 Fluorescent images showing effects of the intraperitoneal administration of saline on Fos expression in the LS of WT and TRPM8 KO mice. The number of Fos^+ nuclei was low in both WT and TRPM8 KO mice. cc, corpus callosum; LV, lateral ventricle. Scale bar = 100 μm.

Supplementary Figure 9

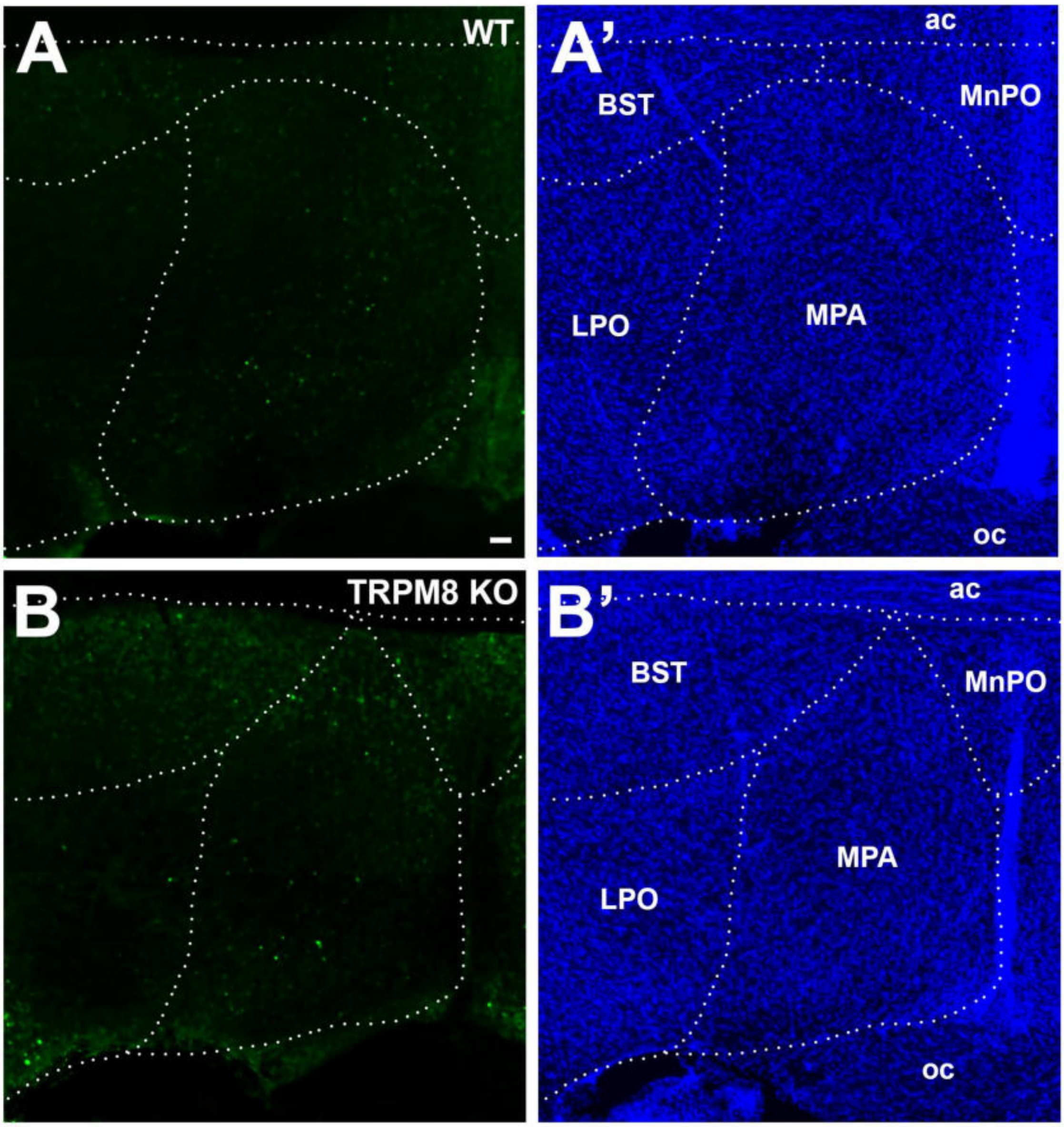

Fig. S9 Fluorescent images showing effects of the intraperitoneal administration of saline on Fos expression in the POA of WT and TRPM8 KO mice. Fos^{+} nuclei were rarely observed in WT and TRPM8 KO mice. ac, anterior commissure; oc, optic chiasma. Scale bar = 100 μm.

Supplementary Figure 10

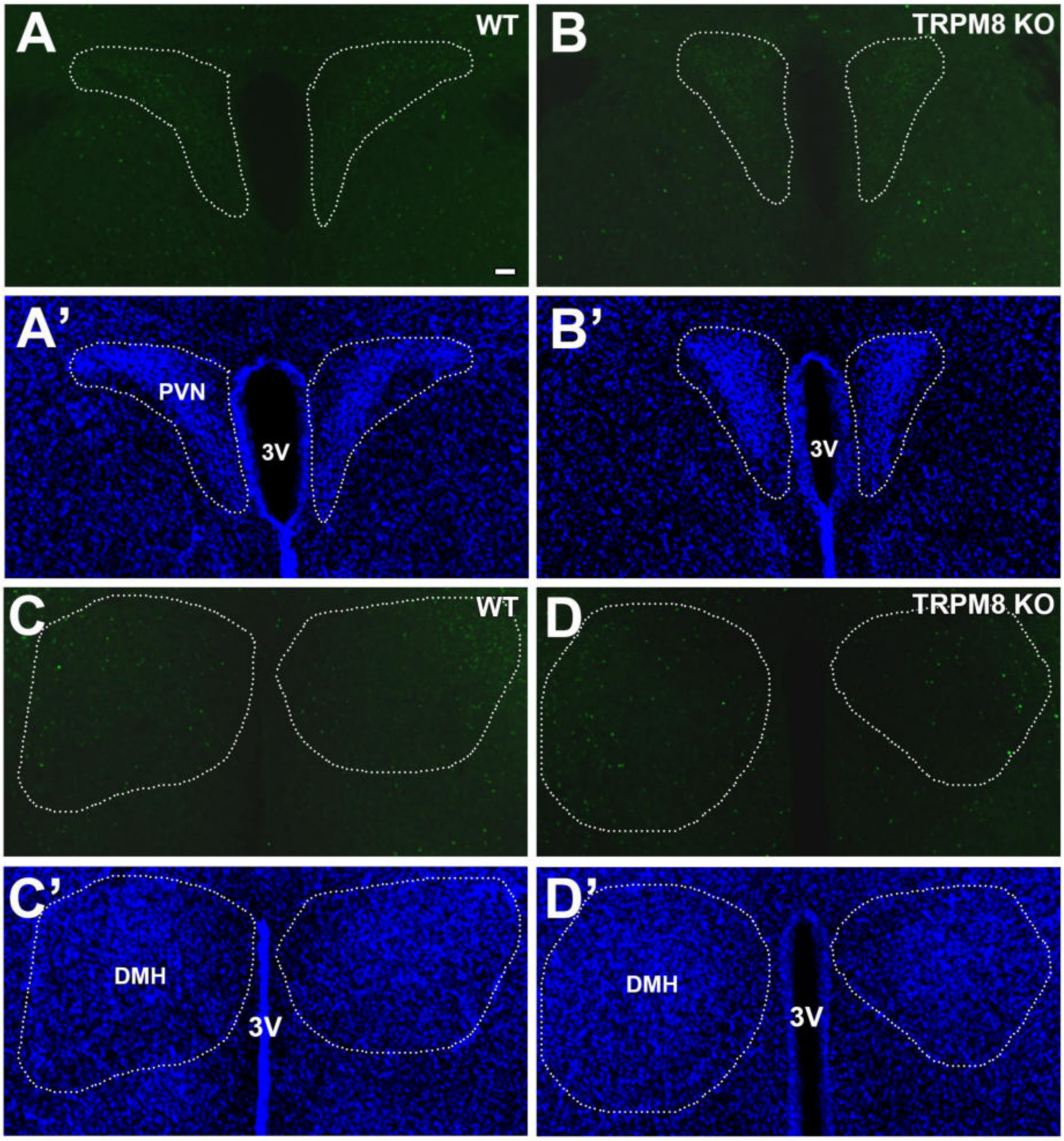

Fig. S10 Fluorescent images showing effects of the intraperitoneal administration of saline on Fos expression in the PVN and DMH of WT and TRPM8 KO mice. Fos^+ nuclei were rarely observed in WT and TRPM8 KO mice. 3V, 3rd ventricle. Scale bar = 100 μm.
